# On the Isoperimetric and Isodiametric Inequalities and the Minimisation of Eigenvalues of the Laplacian

**DOI:** 10.1007/s12220-024-01887-0

**Published:** 2025-01-04

**Authors:** Sam Farrington

**Affiliations:** Department of Mathematical Sciences, Durham University, Mathematical Sciences and Computer Science Building, Upper Mountjoy Campus, Stockton Road, Durham, DH1 3LE UK

**Keywords:** Spectral shape optimisation, Weyl’s law, Isoperimetric inequality, Isodiametric inequality, Mixed boundary conditions, 49R05, 49Q10, 35J25, 35P15

## Abstract

We consider the problem of minimising the *k*-th eigenvalue of the Laplacian with some prescribed boundary condition over collections of convex domains of prescribed perimeter or diameter. It is known that these minimisation problems are well-posed for Dirichlet eigenvalues in any dimension $d\ge 2$ and any sequence of minimisers converges to the ball of unit perimeter or diameter respectively as $k\rightarrow +\infty$. In this paper, we show that the same is true in the case of Neumann eigenvalues under diameter constraint in any dimension and under perimeter constraint in dimension $d=2$. We also consider these problems for Robin eigenvalues and mixed Dirichlet–Neumann eigenvalues, under an additional geometric constraint.

## Introduction

Given $\Omega \subset \mathbb {R}^{d}$ a bounded convex domain, it is well-known that the Dirichlet $-\Delta _{\Omega }^{D}$ and Neumann $-\Delta _{\Omega }^{N}$ Laplacians acting on $\mathcal {L}^{2}(\Omega )$ have discrete spectra, each consisting of a sequence of eigenvalues accumulating only at $+\infty$. We denote the Dirichlet eigenvalues by

$$\begin{aligned} 0< \lambda _{1}(\Omega ) < \lambda _{2}(\Omega ) \le \lambda _{3}(\Omega ) \le \cdots \uparrow + \infty \end{aligned}$$

and the Neumann eigenvalues by

$$\begin{aligned} 0 = \mu _{1}(\Omega ) < \mu _{2}(\Omega ) \le \mu _{3}(\Omega ) \le \cdots \uparrow + \infty . \end{aligned}$$

Moreover, it is well-known that these eigenvalues obey Weyl’s law, see for example [19, §3.2$-$3.3], which asserts that

1 $$\begin{aligned} \lambda _{k}(\Omega ) \sim \mu _{k}(\Omega ) \sim 4\pi ^{2} \left( \frac{k}{\omega _{d}|\Omega |}\right) ^{2/d} =: \frac{W_{d}}{|\Omega |^{2/d}}k^{2/d}, \hspace{5.0pt}\text {as} \hspace{5.0pt}k\uparrow +\infty , \end{aligned}$$

where $|\Omega |$ is the *d*-dimensional volume of $\Omega$ and $\omega _{d}$ is the volume of the *d*-dimensional unit ball. From Weyl’s law, if one knows either the entire Dirichlet spectrum or the entire Neumann spectrum of $\Omega$, then one can determine the volume of $\Omega$.

Naïvely, Weyl’s law and the isoperimetric/isodiametric inequality together suggest that if one minimises either Dirichlet or Neumann eigenvalues over the collection of bounded convex domains of a given perimeter/diameter then for large *k* minimisers should be close to the ball, i.e. the domain with the largest volume. To be clear, by perimeter here we mean the $(d-1)$-dimensional Hausdorff measure of the boundary $\partial \Omega$, which we denote by $|\partial \Omega |$.

In this vein, one can consider the four following spectral shape optimisation problems:

2 $$\begin{aligned} \inf \left\{ \lambda _{k}(\Omega ): \Omega \subset \mathbb {R}^{d} \; \text {bounded, convex}, \; |\partial \Omega | = 1 \right\} , \end{aligned}$$

3 $$\begin{aligned} \inf \left\{ \lambda _{k}(\Omega ): \Omega \subset \mathbb {R}^{d} \; \text {bounded, convex}, \; \textrm{diam}(\Omega ) = 1 \right\} , \end{aligned}$$

4 $$\begin{aligned} \inf \left\{ \mu _{k}(\Omega ): \Omega \subset \mathbb {R}^{d} \; \text {bounded, convex}, \; |\partial \Omega | = 1 \right\} , \end{aligned}$$

5 $$\begin{aligned} \inf \left\{ \mu _{k}(\Omega ): \Omega \subset \mathbb {R}^{d} \; \text {bounded, convex}, \; \textrm{diam}(\Omega ) = 1 \right\} . \end{aligned}$$

For each of the above problems we will discuss when one has existence of minimisers and, if so, the geometric behaviour of minimisers as $k\rightarrow +\infty$. To do this we need to introduce a notion of convergence onto the collection of bounded convex domains. We use the Hausdorff metric here, which is defined as

6 $$\begin{aligned} d_{H}(\Omega _{1},\Omega _{2}):= \max \left\{ \sup _{x\in \Omega _{1}} \inf _{y\in \Omega _{2}} \Vert x-y\Vert _{2}, \sup _{x\in \Omega _{2}}\inf _{y\in \Omega _{1}} \Vert x-y\Vert _{2}\right\} \end{aligned}$$

for two bounded convex domains $\Omega _{1},\Omega _{2} \subset \mathbb {R}^{d}$, where $\Vert \cdot \Vert _{2}$ is the standard Euclidean norm. In this paper, the Hausdorff convergence of bounded convex domains will be taken up to rigid transformations where necessary, i.e $\Omega _{n}$ Hausdorff converges to $\Omega$ as $n\rightarrow +\infty$ if there exists a sequence of rigid transformations $R_{n}:\mathbb {R}^{d}\rightarrow \mathbb {R}^{d}$ such that $d_{H}(R_{n}(\Omega _{n}),\Omega ) \rightarrow 0$ as $n\rightarrow +\infty$. As the Dirichlet and Neumann eigenvalues of a domain are invariant under rigid transformations, this causes no problems.

### Dirichlet Eigenvalue Minimisation

The shape optimisation problem (2) was first considered in the case of $k=1$ and for planar domains by Courant in [16], where it was shown that the ball is the minimiser. This can also be shown using the Faber–Krahn [25, Thm. 3.2.1.] and isoperimetric inequalities. The existence of a minimiser to (2) for any $k\in \mathbb {N}$ when $d = 2$ was given by van den Berg and Iversen in [10] under the more general conditions of minimising the *k*-th Dirichlet eigenvalue among non-empty bounded open sets of unit perimeter without any convexity or connectivity constraints. They showed that a minimiser in this case is necessarily convex. Bucur and Freitas in [6] later showed, when $d=2$, that any sequence $\Omega _{k}^{*}$ of minimisers to (2) Hausdorff converges to the ball of unit perimeter as $k \rightarrow +\infty$. For $d\ge 3$, van den Berg in [5] deduced existence of minimisers and the same asymptotic behaviour as in the two-dimensional case.

#### Theorem 1.1

[5, Thm. 1, adapted] For any $d\ge 2$, there exists a minimiser to (2) for all $k\ge 1$. Moreover, any sequence of minimisers Hausdorff converges to the ball of unit perimeter as $k\rightarrow +\infty$.

In the same paper, [5], van den Berg also considered the shape optimisation problem (3) and deduced an analogous result to Theorem 1.1 in this case.

#### Theorem 1.2

[5, Thm. 1, adapted] For any $d\ge 2$, there exists a minimiser to (3) for all $k\ge 1$. Moreover, any sequence of minimisers Hausdorff converges to the ball of unit diameter as $k\rightarrow +\infty$.

Bogosel, Henrot and Lucardesi also studied the shape optimisation problem (3) in [8] and deduced that the ball is only a minimiser for finitely many $k \in \mathbb {N}$ and minimisers are necessarily bodies of constant width.

### Neumann Eigenvalue Minimisation

In light of Theorems 1.1 and 1.2, the aforementioned naïve notion of minimisers for large *k* being close to a ball holds for Dirichlet eigenvalues. However, for the case of Neumann eigenvalues, the minimisation problem (4) is well-known to be ill-posed for any $k\ge 2$ and $d\ge 3$ as the infimum is zero in this case but $\mu _{2}(\Omega ) > 0$ for any bounded convex domain $\Omega$. Hence, the naïve philosophy that motivated these questions at the beginning of this paper fails here. The fact that this infimum is zero can easily be deduced by considering the sequence of cuboids

$$\begin{aligned} (0,\varepsilon ) \times \cdots \times (0,\varepsilon ) \times \left( 0,\tfrac{1}{2(d-1)}(\varepsilon ^{2-d}-2\varepsilon )\right) \subset \mathbb {R}^{d} \end{aligned}$$

as $\varepsilon \downarrow 0$. When $d=2$, it was shown by van den Berg et al. in [3, Thm. 3.2.] that the minimisation problem

$$\begin{aligned} \inf \left\{ \mu _{k}(\Omega ): \Omega \subset \mathbb {R}^{2} \hspace{5.0pt}\text {rectangle}, \, |\partial \Omega | = 1 \right\} \end{aligned}$$

has a minimiser for all $k\ge 3$ and any sequence of minimisers Hausdorff converges to the square of unit perimeter as $k\rightarrow +\infty$. The analogous result is true in the more general setting of planar convex domains as we now state.

#### Theorem 1.3

When $d=2$, for each $k\ge 3$ there exists a minimiser to (4). Moreover, any sequence of minimisers Hausdorff converges to the ball of unit perimeter as $k\rightarrow +\infty$.

Our methods in this paper are asymptotic in nature and, in fact, only assert that minimisers exist for *k* sufficiently large. It has been shown that they exist for each $k\ge 3$, as in the statement of Theorem 1.3, and not for $k=2$ in [9, Thm. 2.5].

In contrast to the perimeter case, we have that for any $k \ge 2$ and $d\ge 2$ the infimum in (5) is non-zero from the Payne–Weinberger inequality, see [4] and [38], which asserts for any bounded convex domain $\Omega \subset \mathbb {R}^{d}$

7 $$\begin{aligned} \mu _{2}(\Omega ) > \frac{\pi ^{2}}{\textrm{diam}(\Omega )^{2}}. \end{aligned}$$

This inequality is sharp and attained in the limit by the sequence of cuboids

$$\begin{aligned} (0,\varepsilon ) \times \cdots \times (0,\varepsilon ) \times \left( 0,\sqrt{1-(d-1)\varepsilon ^{2}}\right) \subset \mathbb {R}^{d} \end{aligned}$$

as $\varepsilon \downarrow 0$, in the case where $\textrm{diam}(\Omega ) = 1$. It is not immediately clear if and when the shape optimisation problem (5) admits minimisers. However, minimisers do eventually exist for *k* sufficiently large and one can obtain the asymptotic behaviour of minimisers as $k\rightarrow +\infty$. In particular, in dimension two, as in the perimeter case it was shown in [9, Thm. 2.4] that minimisers exist for all $k\ge 3$.

#### Theorem 1.4

For any $d\ge 2$, there exists a constant $N_{d} \in \mathbb {N}$ such that for all $k\ge N_{d}$ there exists a minimiser to (5). Moreover, any sequence $\Omega _{k}^{*}$ of minimisers Hausdorff converges to the ball of unit diameter as $k\rightarrow +\infty$.

The proofs of Theorems 1.3 and 1.4 rest on proving a suitably good family of upper bounds for the Neumann eigenvalue counting function

8 $$\begin{aligned} \mathcal {N}_{\Omega }^{N}(\alpha ):= \# \lbrace k \in \mathbb {N}: \mu _{k}(\Omega ) < \alpha \rbrace \end{aligned}$$

for a bounded convex domain $\Omega \subset \mathbb {R}^{d}$, see Proposition 2.3. We also give a family of lower bounds on the Dirichlet eigenvalue counting function

9 $$\begin{aligned} \mathcal {N}_{\Omega }^{D}(\alpha ):= \# \lbrace k \in \mathbb {N}: \lambda _{k}(\Omega ) < \alpha \rbrace , \end{aligned}$$

from which one can prove a statement of Weyl’s law for sequences bounded convex domains with geometric control, which we will discuss in Sect. 4.

The family of upper bounds on the Neumann counting function can also be used to study a variation of (4) for which one does have the existence of minimisers and for which any sequence of minimisers Hausdorff converges to the ball of unit perimeter as $k\rightarrow +\infty$. The philosophy is if we don’t allow the domains in the collection under consideration to grow too quickly with *k* in terms of their diameter then we obtain non-degenerate asymptotic behaviour. The case $d=2$ is not interesting in this case as two-dimensional convex sets of a fixed perimeter have uniformly bounded diameter.

Before stating the next theorem, for functions $f,g:\mathbb {N} \rightarrow \mathbb {R}_{>0}$, let us remark that the notation $f(k) \ll g(k)$ means that $\limsup _{k\rightarrow +\infty } f(k)/g(k) = 0$ and $f(k) \lesssim g(k)$ means that $\limsup _{k\rightarrow +\infty } f(k)/g(k) <+\infty$ throughout this paper.

#### Theorem 1.5

For any $d\ge 3$ and any $f: \mathbb {N} \rightarrow \mathbb {R}_{>0}$ with $1\ll f(k) \ll k^{1/d(d-1)}$, there exists a constant $N_{d,f} \in \mathbb {N}$ such that for all $k\ge N_{d,f}$ there exists a minimiser to

$$\begin{aligned} \inf \left\{ \mu _{k}(\Omega ): \Omega \subset \mathbb {R}^{d} \hspace{5.0pt}\text {bounded convex domain}, \, |\partial \Omega | \le 1,\, \textrm{diam}(\Omega ) \le f(k)\right\} . \end{aligned}$$

Moreover, any sequence $\Omega _{k}^{*}$ of minimisers Hausdorff converges to the ball of unit perimeter as $k\rightarrow +\infty$.

### Other Related Problems

The methods in this paper can be applied to other spectral shape optimisation problems to obtain asymptotic results.

In [12], the authors study the so-called ‘interior problem’

$$\begin{aligned} \inf \lbrace \mu _{k}(\Omega ): \Omega \subset D, \, \Omega \; \text {convex domain} \rbrace , \end{aligned}$$

where *D* is a fixed bounded convex domain in $\mathbb {R}^{2}$. They deduce necessary and sufficient conditions for the existence of minimisers to this problem. Following a similar strategy to the proof of Theorem 1.4 one can deduce the following result.

#### Theorem 1.6

Given a bounded convex domain $D \subset \mathbb {R}^{d}$, there exists a constant $N_{D}\in \mathbb {N}$ such that for all $k\ge N_{D}$ there exists a minimiser to

$$\begin{aligned} \inf \lbrace \mu _{k}(\Omega ): \Omega \subset D, \, \Omega \; \text {convex domain} \rbrace . \end{aligned}$$

Moreover, any sequence of minimisers Hausdorff converges to *D* as $k\rightarrow +\infty$.

We can also prove results for spectral shape optimisation problems for eigenvalues of the Robin Laplacian $-\Delta _{\Omega ,\beta }^{R}$ with positive Robin parameter $\beta >0$ acting on $\mathcal {L}^{2}(\Omega )$. As in the case of the Dirichlet and Neumann Laplacians, it is well-known that $-\Delta _{\Omega ,\beta }^{R}$ has a discrete collection of eigenvalues accumulating only at $+\infty$ which we denote by

$$\begin{aligned} 0 < \lambda _{1}^{\beta }(\Omega ) \le \lambda _{2}^{\beta }(\Omega ) \le \lambda _{3}^{\beta }(\Omega ) \le \cdots \uparrow +\infty . \end{aligned}$$

Moreover, these eigenvalues satisfy the Dirichlet–Neumann bracketing inequality

$$\begin{aligned} \mu _{k}(\Omega ) \le \lambda _{k}^{\beta }(\Omega ) \le \lambda _{k}(\Omega ), \end{aligned}$$

see for example [30, Thm. 3.2.9.]. And so the Robin eigenvalues also satisfy Weyl’s law, see (1). Due to this bracketing and properties of Robin eigenvalues, from our Neumann eigenvalue counting function bounds we can prove the following two theorems.

#### Theorem 1.7

Fix $\beta \in (0,+\infty )$. For each $k\ge 1$ there exists a minimiser to

$$\begin{aligned} \inf \left\{ \lambda _{k}^{\beta }(\Omega ): \Omega \subset \mathbb {R}^{2} \; \text {bounded, convex}, \; |\partial \Omega | = 1 \right\} . \end{aligned}$$

Moreover, any sequence of minimisers Hausdorff converges to the ball of unit perimeter as $k\rightarrow +\infty$.

#### Remark 1.8

It is unclear to the author if one would expect Theorem 1.7 to hold in higher dimensions. Existence of minimisers for all $k\ge 1$ in any dimension can be shown using Theorem 4.4 in [27] and the lower semi-continuity of Robin eigenvalues under Hausdorff convergence of convex domains, see for example [15, Prop. 3.1]. However, the asymptotic behaviour of minimisers is not known as far as the author of this paper is aware. We note that the conclusion of Theorem 1.5 holds in the case of Robin eigenvalues with positive parameter $\beta > 0$ with existence of minimisers for all $k\ge 1$.

#### Theorem 1.9

Fix $\beta \in (0,+\infty )$. For each $k\ge 1$ there exists a minimiser to

$$\begin{aligned} \inf \left\{ \lambda _{k}^{\beta }(\Omega ): \Omega \subset \mathbb {R}^{d} \; \text {bounded, convex}, \; \textrm{diam}(\Omega ) = 1 \right\} . \end{aligned}$$

Moreover, any sequence of minimisers Hausdorff converges to the ball of unit diameter as $k\rightarrow +\infty$.

Other eigenvalues which satisfy Dirichlet–Neumann bracketing are so-called Zaremba eigenvalues, which satisfy a Neumann boundary condition on part of $\partial \Omega$ and a Dirichlet boundary condition on its complement. In Sect. 5, we study the perimeter constraint eigenvalue minimisation problem for Zaremba eigenvalues in which the shape optimisation problem exhibits the same behaviour as that of (2), see Theorem 1.1. For this we introduce an additional geometric constraint on the collection of convex domains which yields a canonical way of prescribing the mixed boundary conditions and allows us to obtain eigenvalue bounds. Due to the added technicalities in defining this problem, we defer further exposition for later on in the paper.

### Volume Constraint and Other Spectral Functionals

The asymptotic behaviour of optimisers to spectral shape optimisation problems has also been studied in other contexts for differing geometric constraints and spectral functionals. Here we give a brief overview of some related results and remarks on their differences to our own here.

Let $\mathcal {Q}_{d}$ denote the space of *d*-dimensional cuboids, that is the space of all sets of the form $(0,a_{1})\times \cdots \times (0,a_{d})$, $a_{1},\ldots , a_{d} \in (0,+\infty )$, up to a rigid transformation. For such domains one can gain a very strong control on the Dirichlet and Neumann counting functions owing to the fact that they may be written as lattice point counting problems, see [33] for a good overview. This strong control has been utilised to prove results in asymptotic spectral shape optimisation, namely the following was proven by Gittins and Larson in [22].

#### Theorem 1.10

[22, Adapted from Thms 1.1 & 1.2] Let $d\ge 2$.

- For any $k\ge 1$ there exists a minimiser $R_{k}^{*}$ to
  $$\begin{aligned} \inf \lbrace \lambda _{k}(R): R \in \mathcal {Q}_{d},\, |R| = 1\rbrace . \end{aligned}$$
  Moreover, any sequence $R_{k}^{*}$ of minimisers Hausdorff converges to the *d*-dimensional unit cube as $k\rightarrow +\infty$.
- For any $k\ge 1$ there exists a maximiser $S_{k}^{*}$ to
  $$\begin{aligned} \sup \lbrace \mu _{k}(S): S \in \mathcal {Q}_{d},\, |S| = 1\rbrace . \end{aligned}$$
  Moreover, any sequence $S_{k}^{*}$ of maximisers Hausdorff converges to the *d*-dimensional unit cube as $k\rightarrow +\infty$.

One should remark that prior to the work of Gittins and Larson, the above result was known in the Dirichlet case in dimension two [1] and in dimension three [7] and in the Neumann case in dimension two [3]. It is also worth noting the above theorem can be proven using the results of Marshall in [33].

In Theorem 1.10, under volume constraint one maximises Neumann eigenvalues rather than minimises them. This in contrast to Theorems 1.3, 1.4 and 1.5 where we minimise Neumann eigenvalues under perimeter and diameter constraint. Under volume constraint, we have that

$$\begin{aligned} \inf \lbrace \mu _{k}(\Omega ): \Omega \subset \mathbb {R}^{d} \text { bounded convex},\, |\Omega | = 1\rbrace = 0 \end{aligned}$$

for any $k\ge 1$ and any $d\ge 2$ and so the minimisation problem is ill-posed. The fact that the above infimum is zero can be seen by considering the sequence of cuboids

$$\begin{aligned} (0,\varepsilon ^{1-d}) \times (0,\varepsilon ) \times \cdots \times (0,\varepsilon ) \subset \mathbb {R}^{d}. \end{aligned}$$

The philosophy behind wanting to minimise Dirichlet eigenvalues and maximise Neumann eigenvalues under volume constraint comes from the conjectured two-term Weyl asymptotic formula which states that

$$\begin{aligned} \mathcal {N}_{\Omega }^{D}(\alpha ) = \frac{|\Omega |}{(2\pi )^{d}}\omega _{d}\alpha ^{d/2} - \frac{|\partial \Omega |}{4\cdot (2\pi )^{d-1}}\omega _{d-1}\alpha ^{(d-1)/2} + o\big (\alpha ^{(d-1)/2}\big ), \\ \mathcal {N}_{\Omega }^{N}(\alpha ) = \frac{|\Omega |}{(2\pi )^{d}}\omega _{d}\alpha ^{d/2} + \frac{|\partial \Omega |}{4\cdot (2\pi )^{d-1}}\omega _{d-1}\alpha ^{(d-1)/2} + o\big (\alpha ^{(d-1)/2}\big ) \end{aligned}$$

as $\alpha \rightarrow +\infty$. The conjecture is known to hold when $\Omega$ is smooth and satisfies a certain dynamical condition, see [26]. Note that the two-term asymptotic formula suggests that one wants to minimise perimeter in order to minimise large Dirichlet eigenvalues and maximise large Neumann eigenvalues.

In this paper, our control on the Neumann and Dirichlet counting functions is not good enough to obtain asymptotic results concerning the problems

10 $$\begin{aligned} \inf \lbrace \lambda _{k}(\Omega ): \Omega \subset \mathbb {R}^{d} \text { bounded convex},\, |\Omega | = 1\rbrace ,\nonumber \\ \sup \lbrace \mu _{k}(\Omega ): \Omega \subset \mathbb {R}^{d} \text { bounded convex},\, |\Omega | = 1\rbrace . \end{aligned}$$

Moreover, as far as the author is aware, the asymptotic behaviour of optimisers to these problems is unknown. However, extremal problems under volume constraint for averages of eigenvalues have been considered in the literature.

In [20], Freitas considers extremal problems for the average of the first *k* Dirichlet eigenvalues under volume constraint, and also perimeter constraint. Due to the relevance to the results of this paper, we also note that in the perimeter case Freitas proves that any sequence of the associated minimisers Hausdorff converges to the ball as $k\rightarrow +\infty$. Freitas also discusses the analogues of these problems for the average of the first *k* Neumann eigenvalues in Sect. 5 of [20].

Riesz means of eigenvalues have also been studied and results concerning the asymptotic behaviour of optimisers to a problem similar to (10) have been obtained. For $\gamma \ge 0$ we define the Dirichlet Riesz mean by

$$\begin{aligned} \mathcal {R}_{\Omega }^{D,\gamma }(\Lambda ):= \sum _{k:\lambda _{k}(\Omega ) < \Lambda } (\Lambda -\lambda _{k}(\Omega ))^{\gamma } \end{aligned}$$

The Riesz mean $\mathcal {R}_{\Omega }^{D,\gamma }(\Lambda )$ can be viewed as an average of the Dirichlet eigenvalue counting function $\mathcal {N}_{\Omega }^{D}$ given in (9). Note that for $\gamma = 0$, $\mathcal {R}_{\Omega }^{D,\gamma } = \mathcal {N}_{\Omega }^{D}$. Moreover, due to this, minimising Dirichlet eigenvalues is morally the same idea as maximising the Riesz mean.

It was shown in [17, Cor. 1.3] that for any $\gamma \ge 1$ fixed, there exists a maximiser $\Omega _{\gamma ,\Lambda }^{*}$ to

$$\begin{aligned} \sup \lbrace \mathcal {R}_{\Omega }^{D,\gamma }(\Lambda ):\Omega \subset \mathbb {R}^{d} \text { bounded convex},\, |\Omega | = 1\rbrace \end{aligned}$$

for all $\Lambda > 0$. Moreover, letting $\Omega _{\gamma ,\Lambda }^{*}$ denote any choice of such maximiser, one has that $\Omega _{\gamma ,\Lambda }^{*}$ Hausdorff converges to the ball of unit volume as $\Lambda \rightarrow +\infty$.

This fits with the idea that in the regime of volume constraint, one wants to minimise perimeter to minimise large Dirichlet eigenvalues. For further recent results on Riesz means and their associated asymptotic spectral shape optimisation, we refer the reader to [18] and [29].

**Plan of the paper** In Sect. 2, we prove upper bounds on the Neumann eigenvalue counting function and lower bounds on the Dirichlet eigenvalue counting function for bounded convex domains. Using these bounds, we prove Theorems 1.3, 1.4 and 1.5, 1.6, 1.7 and 1.9 in Sect. 3. In Sect. 4, we discuss applications of these bounds with regards to the geometric stability of Weyl’s law. In Sect. 5, we consider some cases where one mixes Dirichlet and Neumann boundary conditions.

## Bounding Dirichlet and Neumann Counting Functions

For a bounded convex domain $\Omega \subset \mathbb {R}^{d}$, recall the definition of its Neumann eigenvalue counting function $\mathcal {N}_{\Omega }^{N}$ from (8) and its Dirichlet eigenvalue counting function $\mathcal {N}_{\Omega }^{D}$ from (9). We aim to prove a suitably good family of upper bounds on $\mathcal {N}_{\Omega }^{N}$, which in turn give us lower bounds on the Neumann eigenvalues of $\Omega$, and a suitably good family of lower bounds on $\mathcal {N}_{\Omega }^{D}$, which in turn give us upper bounds on the Dirichlet eigenvalues of $\Omega$.

In order to prove these bounds, we need to divulge briefly into some convex geometry. We remark that all the results in convex geometry stated in this paper are generally stated for compact convex sets but we have given the equivalent statement here for bounded convex domains for simplicity of exposition. A well-known result in this area is the Minkowski–Steiner theorem, which gives an expression for the volume of a bounded convex domain which is obtained by taking the Minkowski sum of a bounded convex domain $\Omega$ with a ball of radius $\delta > 0$, in terms of geometric quantities associated with $\Omega$ and $\delta > 0$.

From here forward, let $\mathcal {O}_{d}$ denote the collection of bounded convex domains endowed with the Hausdorff topology induced by the metric given by (6).

### Theorem 2.1

(Minkowski–Steiner, see [24, §6]) Let $\Omega$ be a *d*-dimensional convex domain and $\delta > 0$. Then there exist continuous maps $s_{2},s_{3},\ldots ,s_{d-1}: \mathcal {O}_{d} \rightarrow \mathbb {R}$ called the quermassintegrals of $\Omega$ such that

$$\begin{aligned} |\Omega +\delta \mathbb {B}_{d}| = |\Omega | + |\partial \Omega |\delta + \sum _{j=2}^{d-1} \left( {\begin{array}{c}d\\ j\end{array}}\right) s_{j}(\Omega )\delta ^{j} + \omega _{d}\delta ^{d}, \end{aligned}$$

where $\mathbb {B}_{d}$ is the *d*-dimensional unit ball. In particular, as a map $\mathcal {O}_{d} \times (0,+\infty ) \rightarrow \mathbb {R}$, $|\Omega +\delta \mathbb {B}_{d}|$ is continuous.

It is now worth noting some properties of quermassintegrals. Namely, they are monotone with respect to inclusion, i.e. $s_{j}(\Omega _{1})\le s_{j} (\Omega _{2})$ for all *j* and all $\Omega _{1}\subset \Omega _{2}$, and are continuous in the Hausdorff topology, see [24, §6]. The monotonicity property will allow us to obtain bounds on the Neumann eigenvalue counting function which are monotone with respect to domain inclusion.

In addition to the Minkowski–Steiner theorem, we also require the following estimate concerning the volume of interior tubular neighbourhoods of the boundary for convex domains.

### Lemma 2.2

For any $\Omega \in \mathcal {O}_{d}$, one has that

$$\begin{aligned} |\lbrace x\in \Omega : d(x,\partial \Omega ) \le r \rbrace | \le |\partial \Omega |r \end{aligned}$$

for all $$r> 0$$.

### Proof

This follows from Remark 5.7 in [23], which we now briefly outline. We note that in [23] the authors consider $C^{2}$ bounded convex domains but, as they point out, this result holds more generally for bounded convex domains.

Let

$$\begin{aligned} \rho = \sup \left\{ r > 0: \lbrace x\in \Omega : d(x,\partial \Omega ) \le r \rbrace \ne \Omega \right\} , \end{aligned}$$

or equivalently $\rho$ is the inradius of $\Omega$. By the definition of $\rho$, we have that

$$\begin{aligned} |\lbrace x\in \Omega : d(x,\partial \Omega ) \le r \rbrace | = |\Omega | \end{aligned}$$

for all $r\ge \rho$ and so it suffices to consider what happens for $r<\rho$. From the work of Matheron in [34, §2], we have that

$$\begin{aligned} \frac{\textrm{d}}{\textrm{d} r}|\lbrace x\in \Omega : d(x,\partial \Omega ) \le r \rbrace | = P(r) \end{aligned}$$

for all $0<r<\rho$, where *P*(*r*) is the perimeter of the set $\Omega _{r}:= \lbrace x \in \Omega : d(x,\partial \Omega ) \ge r \rbrace$. One may further note that the set $\Omega _{r}$ is convex and so $P(r) \le |\partial \Omega |$ for all $r> 0$ by the monotonicity of perimeter with respect to the inclusion of convex bounded domains. Hence, one has that

$$\begin{aligned} |\lbrace x\in \Omega : d(x,\partial \Omega ) \le r \rbrace | = \int _{0}^{\min (r,\rho )}\textrm{d}s\, P(s) \le \int _{0}^{r}\textrm{d}s\, |\partial \Omega | = |\partial \Omega |r, \end{aligned}$$

for all $r> 0$ which is the desired result. $\square$

We are now ready to state and prove a family of upper bounds for the Neumann eigenvalue counting function. The proof is originally inspired by the proof of Proposition A.1. in [23], whereby the authors give an upper bound on the Neumann counting function of a bounded $C^{2}$ convex domain. Moreover, one should note that the idea of the proof is very classical and can be attributed back to the proof of Weyl’s law in the book of Courant and Hilbert [13]. Although more general than the bound in [23], our bound is also weaker and less general than others in the literature, see Remark 2.5, but it is more convenient to work with and suffices for the purposes of this paper as our focus is the study of spectral shape optimisation problems. So, we favour it for clarity of exposition.

Before stating our family of bounds, we make a notational remark that $\lceil x\rceil$ means the smallest integer bigger than or equal to $x \in \mathbb {R}$.

### Proposition 2.3

For any $n\in \mathbb {N}$, $\Omega \in \mathcal {O}_{d}$ and $\alpha > 0$,

11 $$\begin{aligned} \mathcal {N}_{\Omega }^{N}(\alpha ) \le \frac{ n|\Omega |}{(\mu _{n+1}^{*})^{d/2}} \alpha ^{d/2} + r_{n}(\Omega ;\alpha ), \end{aligned}$$

where

$$\begin{aligned} \begin{aligned} r_{n}(\Omega ;\alpha )&= \left( \frac{\kappa _{n}}{\sqrt{\mu _{n+1}^{*}}}\right) ^{d-1}\left( 2\kappa _{n}+3 \right) d^{1/2}|\partial \Omega | \alpha ^{(d-1)/2} \\  &\qquad + \sum _{j=2}^{d-1} \left( {\begin{array}{c}d\\ j\end{array}}\right) (4d)^{j/2} \left( \frac{\kappa _{n}}{\sqrt{\mu _{n+1}^{*}}}\right) ^{d-j}s_{j}(\Omega )\alpha ^{(d-j)/2} +(4d)^{d/2}\omega _{d}, \end{aligned} \end{aligned}$$

$\mu _{n+1}^{*}$ denotes the $(n+1)$-th Neumann eigenvalue of the *d*-dimensional unit cube, $s_{j}(\Omega )$ denotes the *j*-th quermassintegral of $\Omega$ from the Minkowski–Steiner formula and $\kappa _{n} = \lceil \pi ^{-1}\sqrt{d\mu _{n+1}^{*}}\,\rceil$. Moreover, the remainder $r_{n}(\Omega ;\alpha )$ is monotone with respect to inclusion of convex domains.

### Proof

Fix $\delta > 0$ and $n \in \mathbb {N}$. For $m \in \mathbb {Z}^{d}$, let $Q_{m,\delta }:= \delta (m+(0,1)^{d})$. Note that

$$\begin{aligned} \mathcal {N}_{Q_{m,\delta }}^{N}(\delta ^{-2}\mu _{n+1}^{*}) \le n \end{aligned}$$

by the definition of the Neumann counting function and the scaling property of Neumann eigenvalues under homothety. Setting $\mathcal {I}_{\delta }:= \lbrace m \in \mathbb {Z}^{d}: Q_{m,\delta } \cap \Omega = Q_{m,\delta }\rbrace$, we immediately see that $\# \mathcal {I}_{\delta } \le \delta ^{-d}|\Omega |$ as for any $m\in \mathcal {I}_{\delta }$ we must have $Q_{m,\delta } \subset \Omega$. Then define $\Omega _{\delta }^{i}:= \bigcup _{m\in \mathcal {I}_{\delta }} Q_{m,\delta }$. Taking $\kappa _{n}\in \mathbb {N}$ as given in the statement of the proposition, let

$$\begin{aligned} \mathcal {J}_{\delta }:= \big \lbrace m \in \mathbb {Z}^{d}: Q_{m,\kappa _{n}^{-1}\delta } \cap \Omega \ne \varnothing , Q_{m,\kappa _{n}^{-1}\delta } \cap \Omega _{\delta }^{i} = \varnothing \big \rbrace , \quad \Omega _{\kappa _{n}^{-1}\delta }^{o}:= \Omega \cap \bigcup _{m\in \mathcal {J}_{\delta }} Q_{m,\delta }. \end{aligned}$$

As $\kappa _{n}$ is a positive integer and by construction, we see that $\Omega _{\delta }^{i} \cap \Omega _{\delta }^{o} = \varnothing$, as $\kappa _{n}^{-1} \delta \mathbb {Z}\supset \delta \mathbb {Z}$, and that $\Omega _{\delta }^{i} \cup \Omega _{\kappa _{n}^{-1}\delta }^{o} = \Omega$ up to a set of measure zero. We now argue that for any $m\in \mathcal {J}_{\delta }$, $Q_{m,\kappa _{n}^{-1}\delta }$ must be a subset of the region

$$\begin{aligned} \partial \Omega _{\delta ,\kappa _{n}}:= \big \lbrace x \in \Omega : d(x,\partial \Omega ) \le (2+\kappa _{n}^{-1}) \delta d^{1/2} \big \rbrace \cup \big \lbrace x \in \mathbb {R}^{d}\backslash \Omega : d(x,\partial \Omega ) \le 2(\kappa _{n})^{-1}\delta d^{1/2} \big \rbrace . \end{aligned}$$

Firstly suppose that

$$\begin{aligned} Q_{m,\kappa _{n}^{-1}\delta } \cap \big \lbrace x \in \Omega : d(x,\partial \Omega ) > (2+\kappa _{n}^{-1}) \delta d^{1/2} \big \rbrace \ne \varnothing . \end{aligned}$$

Then we see that $d(m\kappa _{n}^{-1}\delta ,\partial \Omega ) > 2\delta d^{1/2}$. Now let $m^{*} \in \mathbb {Z}^{d}$ be the unique integer lattice point such that $Q_{m^{*},\delta }\supset Q_{m,\kappa _{n}^{-1}\delta }$. Then one easily sees that $\Vert m\kappa _{n}^{-1} \delta - m^{*} \delta \Vert _{2} \le \delta d^{1/2}$ and so one must have that $d(m^{*}\delta , \partial \Omega ) > \delta d^{1/2}$. But this implies that $m^{*} \in \mathcal {I}_{\delta }$ which implies that $m\not \in \mathcal {J}_{\delta }$ and we have a contradiction. Now suppose that

$$\begin{aligned} Q_{m,\kappa _{n}^{-1}\delta } \cap \big \lbrace x \in \mathbb {R}^{d}\backslash \Omega : d(x,\partial \Omega ) > 2(\kappa _{n})^{-1}\delta d^{1/2} \big \rbrace \ne \varnothing . \end{aligned}$$

Then we have that $d(m\kappa _{n}^{-1}\delta ,\partial \Omega ) > \delta (\kappa _{n})^{-1} d^{1/2}$, but again this contradicts $m\in \mathcal {J}_{\delta }$ as in this case $Q_{m,\kappa _{n}^{-1}\delta } \cap \Omega = \varnothing$. Hence, we must indeed have that $Q_{m,\kappa _{n}^{-1}\delta } \subset \partial \Omega _{\delta ,\kappa _{n}}$ if $m\in \mathcal {J}_{\delta }$.

Using the Minkowski–Steiner formula to approximate the volume of $\partial \Omega _{\delta ,\kappa _{n}}\cap (\mathbb {R}^{d}\backslash \Omega )$ and Lemma 2.2 to estimate the volume of $\partial \Omega _{\delta ,\kappa _{n}}\cap \Omega$, we see that

$$\begin{aligned} |\partial \Omega _{\delta ,\kappa _{n}}| \le \left( 2+3\kappa _{n}^{-1}\right) d^{1/2}|\partial \Omega |\delta + \sum _{j=2}^{d-1} \left( {\begin{array}{c}d\\ j\end{array}}\right) (4d)^{j/2} (\kappa _{n})^{-j} s_{j}(\Omega )\delta ^{j} +(4d)^{d/2}\omega _{d}\delta ^{d}. \end{aligned}$$

We then can immediately deduce a bound on the cardinality of $\mathcal {J}_{\delta }$:

$$\begin{aligned}\begin{aligned} \# \mathcal {J}_{\delta }&\le (\kappa _{n})^{d}\delta ^{-d}|\partial \Omega _{\delta ,\kappa _{n}}| \\&\le \left( 2(\kappa _{n})^{d}+3(\kappa _{n})^{d-1}\right) d^{1/2}|\partial \Omega |\delta ^{-d+1} \\  &\qquad + \sum _{j=2}^{d-1} \left( {\begin{array}{c}d\\ j\end{array}}\right) (4d)^{j/2} (\kappa _{n})^{d-j} s_{j}(\Omega )\delta ^{-d+j}+(4d)^{d/2}\omega _{d}. \end{aligned} \end{aligned}$$

Observing that $Q_{m,\kappa _{n}^{-1}\delta } \cap \Omega$ is convex, by our choice of $\kappa _{n}$ and the Payne–Weinberger inequality, see (7),

$$\begin{aligned} \mu _{2}(Q_{m,\kappa _{n}^{-1}\delta } \cap \Omega ) \ge \delta ^{-2}\mu _{n+1}^{*}, \end{aligned}$$

and so $\mathcal {N}_{Q_{m,\kappa _{n}^{-1}\delta } \cap \Omega }^{N}(\delta ^{-2}\mu _{n+1}^{*}) = 1$. By the variational characterisation of Neumann eigenvalues, see [30, Thm. 3.1.11.], it is straightforward to verify that $\mu _{k}(\Omega _{\delta }^{i} \cup \Omega _{\kappa _{n}^{-1}\delta }^{o}) \le \mu _{k}(\Omega )$ for all $k\in \mathbb {N}$, and so it suffices to bound the Neumann eigenvalue counting function of $\Omega _{\delta }^{i} \cup \Omega _{\kappa _{n}^{-1}\delta }^{o}$. Taking $\delta =\alpha ^{-1/2}(\mu _{n+1}^{*})^{1/2}$ and using the bounds on $\#\mathcal {I}_{\delta }$ and $\#\mathcal {J}_{\delta }$, we see that

$$\begin{aligned} \begin{aligned} \mathcal {N}_{\Omega _{\delta }^{i} \cup \Omega _{\kappa _{n}^{-1}\delta }^{o}}^{N}(\alpha )&\le \sum _{m \in \mathcal {I}_{\delta }} \mathcal {N}_{Q_{m,\delta }}^{N}(\alpha ) + \sum _{m \in \mathcal {J}_{\delta }} \mathcal {N}_{Q_{m,\kappa ^{-1}\delta }\cap \Omega }^{N}(\alpha ) \\&\le \frac{n|\Omega |}{(\mu _{n+1}^{*})^{d/2}}\alpha ^{d/2} + \left( \frac{\kappa _{n}}{\sqrt{\mu _{n+1}^{*}}}\right) ^{d-1}\left( 2\kappa _{n}+3\right) d^{1/2} |\partial \Omega | \alpha ^{(d-1)/2} \\&\quad + \sum _{j=2}^{d-1} \left( {\begin{array}{c}d\\ j\end{array}}\right) (4d)^{j/2} \left( \frac{\kappa _{n}}{\sqrt{\mu _{n+1}^{*}}}\right) ^{d-j}s_{j}(\Omega )\alpha ^{(d-j)/2} + (4d)^{d/2}\omega _{d}, \\&= \frac{n|\Omega |}{(\mu _{n+1}^{*})^{d/2}}\alpha ^{d/2} + r_{n}(\Omega ;\alpha ). \end{aligned} \end{aligned}$$

The monotonicity of quermassintegrals with respect to inclusion of bounded convex domains gives the monotonicity of the remainder $r_{n}$ with respect to inclusion of bound convex domains, which completes the proof. $\square$

### Remark 2.4

To make the upper bound in Proposition 2.3 explicit relies on the computability of the quermassintegrals of the domain. In dimension two, the bound simply reads

### Remark 2.5

In Proposition 2.3, we let the parameter $n \in \mathbb {N}$ be independent of $\alpha > 0$. One could let *n* vary with $\alpha$ and obtain a bound so that the leading term in (11) coincides with $(2\pi )^{-d}\omega _{d}|\Omega |\alpha ^{d/2}$, which is the leading asymptotic term from Weyl’s law. By considering upper bounds on the Neumann counting function of the *d*-dimensional unit cube, one can observe that

$$\begin{aligned} \frac{n}{(\mu _{n+1}^{*})^{d/2}} \le (2\pi )^{-d}\omega _{d} + O\left( \big (\mu _{n+1}^{*}\big )^{-1/2}\right) \end{aligned}$$

as $$n\rightarrow +\infty $$. Moreover,

$$\begin{aligned} \left( \frac{\kappa _{n}}{\sqrt{\mu _{n+1}^{*}}}\right) ^{d-1}\left( 2\kappa _{n}+3 \right) = O\left( \big (\mu _{n+1}^{*}\big )^{1/2}\right) \end{aligned}$$

as $n\rightarrow +\infty$. So, taking $n\sim \left( \frac{|\Omega |\sqrt{\alpha }}{|\partial \Omega |}\right) ^{d/2}$ our upper bound in Proposition 2.3 becomes of the form

$$\begin{aligned} \mathcal {N}_{\Omega }^{N}(\alpha ) \le (2\pi )^{-d}\omega _{d}|\Omega |\alpha ^{d/2} + O\left( \alpha ^{(2d-1)/4}\right) . \end{aligned}$$

This is asymptotically worse than the bound given in Theorem 1.3 in [37] which states that

$$\begin{aligned} \mathcal {N}_{\Omega }^{N}(\alpha ) \le (2\pi )^{-d}\omega _{d}|\Omega |\alpha ^{d/2} + O\left( \alpha ^{(d-1)/2}\log \alpha \right) , \end{aligned}$$

and for much more general domains. With the aforementioned choice of *n* depending on $\alpha$, one may prove Propositions 3.1 and 3.2 with a slight modification to the proofs presented in this paper.^1^ We keep the independence of the parameter *n* from $\alpha$ as a stylistic choice.

One can also play the same game with Dirichlet counting functions and prove a lower bound analogously to the proof of Proposition 2.3. Again, as in Remark 2.5, this bound is by no means optimal but is more convenient for us to work with and suffices for our purposes. A better asymptotic bound in a much more general setting and where one does not have a parameter $n\in \mathbb {N}$ may be found in Theorem 1.8. of [37].

### Proposition 2.6

For any $n\in \mathbb {N}$, $\Omega \in \mathcal {O}_{d}$ and $\alpha > 0$,

$$\begin{aligned} \mathcal {N}_{\Omega }^{D}(\alpha ) \ge \frac{n|\Omega |}{(\lambda _{n}^{*})^{d/2}} \alpha ^{d/2} - \frac{2nd^{1/2}|\partial \Omega |}{(\lambda _{n}^{*})^{(d-1)/2}}\alpha ^{(d-1)/2}, \end{aligned}$$

where $\lambda _{n}^{*}$ is the *n*-th Dirichlet eigenvalue of the *d*-dimensional unit cube.

### Proof

Let $\varepsilon > 0$. For $m\in \mathbb {Z}^{d}$ and $\delta > 0$, define $Q_{m,\delta }$ and $\mathcal {I}_{\delta }$ as in the proof of Proposition 2.3. It is clear that for a given $m\in \mathbb {Z}^{d}$ if $Q_{m,\delta } \cap \lbrace x \in \Omega : d(x,\partial \Omega ) \ge 2\delta d^{1/2} \rbrace \ne \varnothing$ then $m\in \mathcal {I}_{\delta }$. Hence, we obtain that

12 $$\begin{aligned} \# \mathcal {I}_{\delta } \ge \delta ^{-d}| \lbrace x \in \Omega : d(x,\partial \Omega ) \ge 2\delta d^{1/2} \rbrace | \ge \delta ^{-d} |\Omega | - 2d^{1/2} \delta ^{-d+1}|\partial \Omega |. \end{aligned}$$

Noting that $\mathcal {N}_{Q_{m,\delta }}^{D}(\delta ^{-2}(\lambda _{n}^{*}+\varepsilon ))\ge n$, by the variational characterisation of Dirichlet eigenvalues, see [30, Thm. 3.1.9.], it suffices to bound the counting function of $\bigcup _{m\in \mathcal {I}_{\delta }} Q_{m,\delta }$ from below. Hence, taking $\delta = \alpha ^{-1/2}(\lambda _{n}^{*}+\varepsilon )^{1/2}$ and using the estimate on $\# \mathcal {I}_{\delta }$ from (12), we see that

$$\begin{aligned} \mathcal {N}_{\Omega }^{D}(\alpha ) \ge \sum _{m\in \mathcal {I}_{\delta }} \mathcal {N}_{Q_{m,\delta }}^{D}(\alpha ) \ge \frac{n|\Omega |}{(\lambda _{n}^{*}+\varepsilon )^{d/2}} \alpha ^{d/2} - \frac{2nd^{1/2}|\partial \Omega |}{(\lambda _{n}^{*}+\varepsilon )^{(d-1)/2}}\alpha ^{(d-1)/2}. \end{aligned}$$

Taking $\varepsilon \downarrow 0$ completes the proof. $\square$

## Proofs of Theorems 1.3, 1.4, 1.5, 1.6, 1.7 and 1.9

For $d\ge 3$, one can make the upper bound in Proposition 2.3 uniform over a given collection of convex domains provided that the convex domains are all subsets of a larger convex domain. This can be done by constraining the diameter of the domains. This is due to the monotonicity of the remainder in Proposition 2.3 as any convex domain of diameter $D>0$ can be contained in a ball of diameter 2*D*.

We now show how one can construct asymptotic uniform lower bounds on Neumann eigenvalues of convex domains. In fact, we do not need uniform control on the diameter of the convex domains, we only need a certain control for each *k*, as we now prove. Recall that $\mathcal {O}_{d}$ denotes the collection of bounded convex domains endowed with the Hausdorff topology induced by the metric given by (6) and $W_{d}$ denotes the Weyl constant from (1).

### Proposition 3.1

For any $$V >0 $$ and any $$f: \mathbb {N}\rightarrow \mathbb {R}_{>0}$$ such that $$c\le f(k) \ll k^{1/d(d-1)}$$ as $$k\rightarrow +\infty $$ for some $$c > 0$$,

$$\begin{aligned} \liminf _{k\rightarrow +\infty } k^{-2/d} \Big [ \inf \left\{ \mu _{k}(\Omega ): \Omega \in \mathcal {O}_{d}, \, |\Omega | \le V, \, \textrm{diam}(\Omega ) \le f(k) \right\} \Big ] \ge W_{d}V^{-2/d}. \end{aligned}$$

### Proof

Let $k,n\in \mathbb {N}$ and $\varepsilon > 0$ be fixed, and let $\Omega \in \mathcal {O}_{d}$ with $|\Omega |\le V$ and $\textrm{diam}(\Omega ) \le f(k)$. From the bound in Proposition 2.3, using the monotonicity of the remainder, we see that

$$\begin{aligned} k \le \mathcal {N}_{\Omega }^{N}(\mu _{k}(\Omega )+\varepsilon ) \le \frac{n|\Omega |}{(\mu _{n+1}^{*})^{d/2}}(\mu _{k}(\Omega )+\varepsilon )^{d/2} + r_{n}(B_{k};\mu _{k}(\Omega )+\varepsilon ), \end{aligned}$$

where $B_{k}$ is the ball of diameter 2*f*(*k*). Since $\Omega$ was arbitrary, we see that

13 $$\begin{aligned} 1 \le \frac{nV}{k(\mu _{n+1}^{*})^{d/2}}(m_{k}+\varepsilon )^{d/2} + k^{-1}r_{n}(B_{k};m_{k}+\varepsilon ), \end{aligned}$$

where

$$\begin{aligned} m_{k} = \inf \left\{ \mu _{k}(\Omega ): \Omega \in \mathcal {O}_{d}, \, |\Omega | \le V, \, \textrm{diam}(\Omega ) \le f(k) \right\} . \end{aligned}$$

Setting $\overline{m}_{k} = k^{-2/d}(m_{k}+\varepsilon )$, writing out the right-hand side of (13) we see that

$$\begin{aligned} \begin{aligned} 1&\le \frac{nV}{(\mu _{n+1}^{*})^{d/2}}(\overline{m}_{k})^{d/2} + C_{d,n} k^{-1/d}|\partial B_{k}|(\overline{m}_{k})^{(d-1)/2} \\&\qquad + \sum _{j=2}^{d-1} C_{d,n,j}' s_{j}(B_{k}) k^{-j/d}(\overline{m}_{k})^{(d-j)/2} + C_{d}'' k^{-1} \\&:= p_{n,k}(\overline{m}_{k}), \end{aligned} \end{aligned}$$

for some constants $C_{d,n},C_{d,n,j}',C_{d}''>0$ whose dependence is denoted in the subscript. By the scaling properties of quermassintegrals we see that $k^{-1/d}|\partial B_{k}|,k^{-j/d}s_{j}(B_{k}) \rightarrow 0$ as $k\rightarrow +\infty$. Hence, for any $0<\delta <1$, there exists $k_{\delta } \in \mathbb {N}$ such that for all $k \ge k_{\delta }$ we have

$$\begin{aligned} 1 \le p_{n,k}(\overline{m}_{k}) \le \frac{nV}{(\mu _{n+1}^{*})^{d/2}}(\overline{m}_{k})^{d/2} + \delta \sum _{j=1}^{d} (\overline{m}_{k})^{(d-j)/2}. \end{aligned}$$

Let $\gamma _{n,\delta }$ be the unique positive solution to

$$\begin{aligned} \frac{nV}{(\mu _{n+1}^{*})^{d/2}}(\gamma _{n,\delta })^{d/2} + \delta \sum _{j=1}^{d} (\gamma _{n,\delta })^{(d-j)/2} = 1, \end{aligned}$$

then we immediately deduce that $\overline{m}_{k} \ge \gamma _{n,\delta }$ for any $k\ge k_{\delta }$, as $p_{n,k}: (0,+\infty ) \rightarrow \mathbb {R}$ is strictly monotone increasing for each $n,k\in \mathbb {N}$. Since $\delta > 0$ was arbitrary, we see that

$$\begin{aligned} \liminf _{k\rightarrow + \infty }\overline{m}_{k} \ge \lim _{\delta \downarrow 0} \gamma _{n,\delta } = \left( \frac{(\mu _{n+1}^{*})^{d/2}}{nV}\right) ^{2/d} = \frac{\mu _{n+1}^{*}}{n^{2/d}V^{2/d}}. \end{aligned}$$

And so

$$\begin{aligned} \liminf _{k\rightarrow +\infty } k^{-2/d}m_{k} \ge \frac{\mu _{n+1}^{*}}{n^{2/d}V^{2/d}} \end{aligned}$$

as $\varepsilon > 0$ was arbitrary. Taking the limit as $n\rightarrow +\infty$ gives the result by Weyl’s law. $\square$

One can also prove the following result completely analogously to that of Proposition 3.1.

### Proposition 3.2

For any $$V >0 $$ and any $$f: \mathbb {N}\rightarrow \mathbb {R}_{>0}$$ such that $$c\le f(k) \ll k^{1/2}$$ as $$k\rightarrow +\infty $$ for some $$c > 0$$,

$$\begin{aligned} \liminf _{k\rightarrow +\infty } k^{-1} \Big [ \inf \left\{ \mu _{k}(\Omega ): \Omega \in \mathcal {O}_{2}, \, |\Omega | \le V, \, |\partial \Omega | \le f(k) \right\} \Big ] \ge 4\pi V^{-1}. \end{aligned}$$

The proofs of Theorems 1.3, 1.4, 1.51.6, 1.7 and 1.9 now immediately follow from the proof of Propositions 3.1 and 3.2. Before giving the proofs we state a variation of Blaschke’s selection theorem which suffices for our purposes.

### Lemma 3.3

(Blaschke’s selection theorem) Any sequence $\Omega _{n} \in \mathcal {O}_{d}$ with $|\Omega _{n}| \ge C_{1}$ and $\textrm{diam}(\Omega _{n}) \le C_{2}$ for all $n\in \mathbb {N}$ for some positive constants $C_{1},C_{2}>0$ has a Hausdorff convergent subsequence, up to possible translations of elements of the sequence.

### Proof

From Lemma 3 in [5] and the constraints on the volume and diameter of the $\Omega _{n}$, we have that

$$\begin{aligned} \rho (\Omega _{n}) \ge 2^{-d} (d\omega _{d})^{-1} \textrm{diam}(\Omega )^{1-d}|\Omega _{n}| \ge \rho ^{*} \end{aligned}$$

where $\rho (\Omega _{n})$ denotes the inradius of $\Omega _{n}$ and $\rho ^{*}>0$ is a constant. Hence, as the diameters of the $\Omega _{n}$ are uniformly bounded, we can find a suitably large compact convex domain $K'$ such that we can arrange the $\Omega _{n}$ so that

$$\begin{aligned} B(0;\rho ^{*}) \subset \Omega _{n} \subset K' \end{aligned}$$

for each $n\in \mathbb {N}$. Applying the classical form of Blaschke’s selection theorem, see [24, Thm. 6.3], the sequence $\overline{\Omega _{n}}$ has a Hausdorff convergent subsequence $\overline{\Omega _{n_{k}}}$ converging to some compact convex set *K* with non-empty interior as $k\rightarrow +\infty$. Denoting the interior of *K* by $\Omega$, we see that $\Omega _{n_{k}} \rightarrow \Omega$ as $k\rightarrow +\infty$ with respect to the Hausdorff metric which gives the result. $\square$

### Proof of Theorems 1.3 and 1.7

#### Proof of Theorem 1.3

Existence of minimisers for all $k\ge 3$ comes directly from Theorem 2.5 in [9]. So it suffices to prove the asymptotic behaviour of any sequence $\Omega _{k}^{*}$ of minimisers as $k\rightarrow +\infty$. Let $f(k)=1$ and take $V > 0$ to be the volume of the two-dimensional ball of unit perimeter, which we denote by *B*. From Weyl’s law we know that

$$\begin{aligned} \mu _{k}(B) \sim \frac{4\pi k}{V} \end{aligned}$$

and from Proposition 3.2 we see that for any $0< \varepsilon < V$ and $\delta > 0$

$$\begin{aligned} \inf \left\{ \mu _{k}(\Omega ): \Omega \in \mathcal {O}_{2}, \, |\Omega | \le V-\varepsilon , \, |\partial \Omega | = 1 \right\} \ge \frac{4\pi k}{V-\varepsilon } - \delta \end{aligned}$$

for *k* sufficiently large. Combining these two results, we see that for any $0<\varepsilon < V$, for *k* sufficiently large

$$\begin{aligned} \mu _{k}(B) < \inf \left\{ \mu _{k}(\Omega ): \Omega \in \mathcal {O}_{2}, \, |\Omega | \le V-\varepsilon , \, |\partial \Omega | = 1 \right\} . \end{aligned}$$

Hence, one must have that $|\Omega _{k}^{*}| > V-\varepsilon$ for *k* sufficiently large. Since $0< \varepsilon < V$ was arbitrary, we see that $|\Omega _{k}^{*}| \rightarrow V$ as $k\rightarrow +\infty$. Using Bonnesen’s quantitative isoperimetric inequality, see [11, 28], one can deduce that the $\partial \Omega _{k}^{*}$ eventually lie, up to rigid planar motions, inside the annulus

$$\begin{aligned} (\partial B)_{\delta }:= \lbrace x \in \mathbb {R}^{2}: d(x,\partial B) \le \delta \rbrace \end{aligned}$$

for any $\delta >0$ for *k* sufficiently large. Hence, the $\Omega _{k}^{*}$ Hausdorff converge, up to possible rigid planar motions, to *B* as $k\rightarrow +\infty$, which completes the proof. $\square$

#### Proof of Theorem 1.7

We first show that minimisers exist for each $k\ge 1$. Fix $k\ge 1$, using Proposition 2.3 in [2], we see that

$$\begin{aligned} \inf \left\{ \lambda _{k}^{\beta }(\Omega ): \Omega \in \mathcal {O}_{2}, \, |\Omega | \le \varepsilon \right\} \uparrow + \infty \end{aligned}$$

as $\varepsilon \downarrow 0$. Hence, there exists $\varepsilon _{0} > 0$ such that

14 $$\begin{aligned} \inf \left\{ \lambda _{k}^{\beta }(\Omega ): \Omega \in \mathcal {O}_{2}, \, |\partial \Omega | = 1 \right\} = \inf \left\{ \lambda _{k}^{\beta }(\Omega ): \Omega \in \mathcal {O}_{2}, \, |\partial \Omega | = 1, \, |\Omega | \ge \varepsilon _{0} \right\} .\nonumber \\ \end{aligned}$$

By Lemma 3.3 and the inequality $2\textrm{diam}(\Omega ) \le |\partial \Omega |$ for $\Omega \in \mathcal {O}_{2}$, the set on the right-hand side of (14) is sequentially compact, up to possible translation of the elements of a given sequence, with respect to the Hausdorff metric. Thus, any minimising sequence $\Omega _{n}$ in this set has a Hausdorff convergent subsequence, up to translations, which we also denote by $\Omega _{n}$, converging to some $\Omega ^{*} \in \mathcal {O}_{2}$ as $n\rightarrow +\infty$ with $|\partial \Omega ^{*}| =1$ and $|\Omega ^{*}| \ge \varepsilon _{0}$. Hence, using semi-continuity of Robin eigenvalues under Hausdorff convergence of bounded convex domains, see [15, Prop. 3.1.], one obtains that

$$\begin{aligned} \lambda _{k}^{\beta }(\Omega ^{*}) \le \liminf _{n\rightarrow +\infty } \lambda _{k}^{\beta }(\Omega _{n}) = \inf \left\{ \lambda _{k}^{\beta }(\Omega ): \Omega \in \mathcal {O}_{2}, \, |\partial \Omega | = 1 \right\} . \end{aligned}$$

And so minimisers exist for all $k\ge 1$.

The proof of the asymptotic behaviour of minimisers follows completely analogously to the proof of Theorem 1.3 using the inequality $\mu _{k}(\Omega ) \le \lambda _{k}^{\beta }(\Omega )$ and that the Robin eigenvalues $\lambda _{k}^{\beta }(\Omega )$ satisfy Weyl’s law. $\square$

### Proof of Theorems 1.4, 1.5, 1.6 and 1.9

#### Proof of Theorem 1.4

In dimension two, the proof of existence of minimisers for all $k\ge 3$ and not for $k=2$ follows from Theorem 2.4 in [9]. In higher dimensions, we cannot use the same trick as in two-dimensions as collapsing sequences of convex domains of unit diameter do not necessarily collapse to a line segment. Instead we show that minimisers must eventually exist from the asymptotic result in Proposition 3.1.

Let $f(k)=1$ and take $V > 0$ to be the volume of the *d*-dimensional ball of unit diameter, which we denote by *B*. Weyl’s law tells us that

$$\begin{aligned} \mu _{k}(B) \sim \frac{W_{d}}{V^{2/d}}k^{2/d} \end{aligned}$$

and from Proposition 3.1 we see that for any $0<\varepsilon <V$ and $\delta > 0$, for *k* sufficiently large

$$\begin{aligned} \inf \left\{ \mu _{k}(\Omega ): \Omega \in \mathcal {O}_{d}, \, |\Omega | \le V - \varepsilon , \, \textrm{diam}(\Omega ) \le 1 \right\} \ge \frac{W_{d}}{(V-\varepsilon )^{2/d}}k^{2/d}-\delta \end{aligned}$$

Thus, for any $0<\varepsilon <V$, for *k* sufficiently large

15 $$\begin{aligned} \begin{aligned} \mu _{k}(B)&< \inf \left\{ \mu _{k}(\Omega ): \Omega \in \mathcal {O}_{d}, \, |\Omega | \le V - \varepsilon , \, \textrm{diam}(\Omega ) \le 1 \right\} \\&\le \inf \left\{ \mu _{k}(\Omega ): \Omega \in \mathcal {O}_{d}, \, |\Omega | \le V - \varepsilon , \, \textrm{diam}(\Omega ) = 1 \right\} . \end{aligned} \end{aligned}$$

Hence,

16 $$\begin{aligned} \begin{aligned}&\inf \left\{ \mu _{k}(\Omega ): \Omega \in \mathcal {O}_{d}, \, \textrm{diam}(\Omega ) = 1 \right\} \\  &\qquad \qquad = \inf \left\{ \mu _{k}(\Omega ): \Omega \in \mathcal {O}_{d}, \, |\Omega | \ge V -\varepsilon , \, \textrm{diam}(\Omega ) = 1 \right\} . \end{aligned} \end{aligned}$$

By Lemma 3.3, the infimum on the right hand side of (16) is taken over a set which is sequentially compact, up to possible translations of elements of a given sequence. Moreover, Neumann eigenvalues are continuous with respect to Hausdorff convergence of convex domains, see for example [39], and so a simple application of the extreme value theorem shows that a minimiser must necessarily exist for *k* sufficiently large.

As $0<\varepsilon < V$ was arbitrary in (15), it is clear that one necessarily has for any sequence $\Omega _{k}^{*}$ of minimisers, $|\Omega _{k}^{*}|\rightarrow V$ as $k\rightarrow +\infty$. Using the quantitative isodiametric inequality [36, Thm. 1], one can deduce that the $\Omega _{k}^{*}$ necessarily Hausdorff converge, up to possible rigid transformations, to *B* as $k\rightarrow +\infty$, which completes the proof. $\square$

#### Proof of Theorem 1.5

As the assumptions on the function $f: \mathbb {N} \rightarrow (0,+\infty )$ in Theorem 1.5 are the same as those in Proposition 3.1, following the same lines of argument of the proof of Theorem 1.4, and using the quantitative isoperimetric inequality results due to Fuglede in [21], one can prove Theorem 1.5 analogously. $\square$

#### Proof of Theorem 1.6

A simple application of Proposition 3.1 shows that for any $\varepsilon > 0$

17 $$\begin{aligned} \liminf _{k\rightarrow +\infty } k^{-2/d}\Big [ \inf \left\{ \mu _{k}(\Omega ): \Omega \subset D, \, \Omega \; \text {convex domain}, \, |\Omega | \le |D| - \varepsilon \right\} \Big ] \ge \frac{W_{d}}{(|D|-\varepsilon )^{2/d}}. \end{aligned}$$

In the same way as in the proof of Theorem 1.3, one can show by comparing (17) with Weyl’s law for $\mu _{k}(D)$, that minimisers must exist for *k* sufficiently large. Moreover, Weyl’s law for $\mu _{k}(D)$ and (17) also imply that for any sequence of minimisers $\Omega _{k}^{*}$ we have that $|\Omega _{k}^{*}| \rightarrow D$ as $k\rightarrow +\infty$, as $\varepsilon > 0$ was arbitrary. Hence, using Lemma 3.3 and the continuity of volume under Hausdorff convergence of convex domains, one immediately deduces that the $\Omega _{k}^{*}$ must Hausdorff converge to *D* as $k\rightarrow +\infty$. $\square$

#### Proof of Theorem 1.9

We first show that minimisers exist for each $k\ge 1$. Fix $k\ge 1$, using Proposition 2.3 in [2], we see that

$$\begin{aligned} \inf \left\{ \lambda _{k}^{\beta }(\Omega ): \Omega \in \mathcal {O}_{d}, \, |\Omega | \le \varepsilon \right\} \uparrow + \infty \end{aligned}$$

as $\varepsilon \downarrow 0$. Hence, there exists $\varepsilon _{0} > 0$ such that

18 $$\begin{aligned}  &   \inf \left\{ \lambda _{k}^{\beta }(\Omega ): \Omega \in \mathcal {O}_{2}, \, \textrm{diam}(\Omega ) = 1 \right\} \nonumber \\  &   = \inf \left\{ \lambda _{k}^{\beta }(\Omega ): \Omega \in \mathcal {O}_{2}, \, \textrm{diam}(\Omega ) = 1, \, |\Omega | \ge \varepsilon _{0} \right\} . \end{aligned}$$

By Lemma 3.3, the set on the right-hand side of (18) is sequentially compact, up to possible translation of the elements of a given sequence, with respect to the Hausdorff metric. Thus, any minimising sequence $\Omega _{n}$ in this set has a Hausdorff convergent subsequence, up to translations, which we also denote by $\Omega _{n}$, converging to some $\Omega ^{*} \in \mathcal {O}_{d}$ as $n\rightarrow +\infty$ with $\textrm{diam}(\Omega ) =1$ and $|\Omega ^{*}| \ge \varepsilon _{0}$. Hence, using semi-continuity of Robin eigenvalues under Hausdorff convergence of bounded convex domains, see [15, Prop. 3.1.], one obtains that

$$\begin{aligned} \lambda _{k}^{\beta }(\Omega ^{*}) \le \liminf _{n\rightarrow +\infty } \lambda _{k}^{\beta }(\Omega _{n}) = \inf \left\{ \lambda _{k}^{\beta }(\Omega ): \Omega \in \mathcal {O}_{2}, \, |\partial \Omega | = 1 \right\} . \end{aligned}$$

And so minimisers exist for all $k\ge 1$.

The proof of the asymptotic behaviour of minimisers follows completely analogously to the proof of Theorem 1.4 using the inequality $\mu _{k}(\Omega ) \le \lambda _{k}^{\beta }(\Omega )$ and that $\lambda _{k}^{\beta }(\Omega )$ satisfies Weyl’s law. $\square$

## Geometric Stability of Weyl’s Law

In this section, we discuss further applications of the bounds obtained in Sect. 2 to stability results concerning Weyl’s law, see Theorem 4.2. These results will become of use when one wants to obtain asymptotic shape optimisation results for mixed Dirichlet–Neumann eigenvalues in Sect. 5.

Recall again that $\mathcal {O}_{d}$ denotes the collection of bounded convex domains endowed with the Hausdorff topology induced by the metric given by (6) and $W_{d}$ denotes the Weyl constant from (1).

Firstly, we can carry out similar reasoning to that in the last section to yield asymptotic uniform upper bounds for Dirichlet eigenvalues.

### Proposition 4.1

For any $$V >0 $$ and any $$f: \mathbb {N}\rightarrow \mathbb {R}_{>0}$$ such that $$f(k)\ll k^{1/d}$$ as $$k\rightarrow +\infty $$,

$$\begin{aligned} \limsup _{k\rightarrow +\infty } k^{-2/d} \Big [ \sup \left\{ \lambda _{k}(\Omega ): \Omega \in \mathcal {O}_{d}, \, |\Omega | \ge V, \, |\partial \Omega | \le f(k) \right\} \Big ] \le W_{d}V^{-2/d} \end{aligned}$$

as $k\rightarrow +\infty$, provided that the set

$$\begin{aligned} \left\{ \lambda _{k}(\Omega ): \Omega \in \mathcal {O}_{d}, \, |\Omega | \ge V, \, |\partial \Omega | \le f(k) \right\} \end{aligned}$$

remains non-empty.

### Proof

For an arbitrary $\Omega \in \mathcal {O}_{d}$ with $|\Omega | \ge V$ and $|\partial \Omega | \le f(k)$, observe that

$$\begin{aligned} \begin{aligned} k \ge \mathcal {N}_{\Omega }^{D}(\lambda _{k}(\Omega ))&\ge \frac{n|\Omega |}{(\lambda _{n}^{*})^{d/2}} \lambda _{k}(\Omega )^{d/2} - \frac{2nd^{1/2}|\partial \Omega |}{(\lambda _{n}^{*})^{(d-1)/2}}\lambda _{k}(\Omega )^{(d-1)/2} \\&\ge \frac{nV}{(\lambda _{n}^{*})^{d/2}} \lambda _{k}(\Omega )^{d/2} - \frac{2nd^{1/2}f(k)}{(\lambda _{n}^{*})^{(d-1)/2}}\lambda _{k}(\Omega )^{(d-1)/2}, \end{aligned} \end{aligned}$$

using the bound from Proposition 2.6. Setting

$$\begin{aligned} M_{k}:= \sup \left\{ \lambda _{k}(\Omega ): \Omega \in \mathcal {O}_{d}, \, |\Omega | \ge V, \, |\partial \Omega | \le f(k) \right\} , \end{aligned}$$

we have that

19 $$\begin{aligned} k \ge \frac{nV}{(\lambda _{n}^{*})^{d/2}} \lambda _{k}(\Omega )^{d/2} - \frac{2nd^{1/2}f(k)}{(\lambda _{n}^{*})^{(d-1)/2}}(M_{k})^{(d-1)/2} \end{aligned}$$

by the definition of $M_{k}$. Now, by taking the supremum over the RHS of (19) and dividing through by *k*,

20 $$\begin{aligned} 1 \ge \frac{nV}{(\lambda _{n}^{*})^{d/2}} \left( \frac{M_{k}}{k^{2/d}}\right) ^{d/2} - \frac{2nd^{1/2}f(k)}{k^{1/d}(\lambda _{n}^{*})^{(d-1)/2}}\left( \frac{M_{k}}{k^{2/d}}\right) ^{(d-1)/2}. \end{aligned}$$

Since $f(k) \ll k^{1/d}$, we see that $k^{-1/d}f(k)$ is a bounded sequence and so taking $n=1$, we have that there exist constants $C_{1},C_{2}>0$ such that

$$\begin{aligned} C_{1}\left( \frac{M_{k}}{k^{2/d}}\right) ^{d/2}-C_{2}\left( \frac{M_{k}}{k^{2/d}}\right) ^{(d-1)/2} - 1 \le 0. \end{aligned}$$

From which it immediately follows that there exists a constant $C>0$ such that

$$\begin{aligned} M_{k} \le C k^{2/d}. \end{aligned}$$

Now in view of Eq. (20), we have

$$\begin{aligned} 1 \ge \frac{nV}{(\lambda _{n}^{*})^{d/2}} \left( \frac{M_{k}}{k^{2/d}}\right) ^{d/2} - \frac{2nd^{1/2}f(k)}{k^{1/d}(\lambda _{n}^{*})^{(d-1)/2}}C^{(d-1)/2}, \end{aligned}$$

Taking the limsup as $k\rightarrow +\infty$ and rearranging yields that

$$\begin{aligned} \limsup _{k\rightarrow +\infty } k^{-2/d}M_{k} \le \frac{\lambda _{n}^{*}}{n^{2/d}V^{2/d}}. \end{aligned}$$

Since $n\in \mathbb {N}$ was arbitrary we see that

$$\begin{aligned} \limsup _{k\rightarrow +\infty } k^{-2/d}M_{k} \le W_{d}V^{-2/d} \end{aligned}$$

using Weyl’s law, which completes the proof. $\square$

As a direct consequence of Propositions 3.1 and 4.1 one can deduce the following variation of Weyl’s law for bounded convex domains.

### Theorem 4.2

Let $\Omega _{k}\subset \mathbb {R}^{d}$ be a sequence of bounded convex domains of volume $V > 0$ and $\textrm{diam}(\Omega _{k}) \ll k^{1/(d(d-1))}$ as $k\rightarrow +\infty$, then

21 $$\begin{aligned} \lambda _{k}(\Omega _{k}) \sim \mu _{k}(\Omega _{k}) \sim 4\pi ^{2} \left( \frac{k}{\omega _{d} V}\right) ^{2/d} \end{aligned}$$

as $$k\rightarrow +\infty $$.

### Proof

Noting that the condition $\textrm{diam}(\Omega _{k}) \ll k^{1/(d(d-1))}$ as $k\rightarrow +\infty$ implies that $|\partial \Omega _{k}|\ll k^{1/d}$ as $k\rightarrow +\infty$ and that by classical variational arguments $\mu _{k}(\Omega _{k}) \le \lambda _{k}(\Omega _{k})$, combining a simple application of Propositions 3.1 and 4.1 gives the result. $\square$

The condition $\textrm{diam}(\Omega _{k}) \ll k^{1/(d(d-1))}$ is sharp in the sense that one can construct sequences of domains with $\textrm{diam}(\Omega _{k}) \lesssim k^{1/(d(d-1))}$ for which (21) does not hold. For example, in two dimensions one can consider the sequence of domains $\Omega _{k} = (0,(4k)^{1/2}) \times (0,(4k)^{-1/2})$. The philosophy of Theorem 4.2 is that if we do not allow the geometry of the $\Omega _{k}$ to degenerate too quickly as $k\rightarrow +\infty$ then the leading Weyl term will dominate against both the Dirichlet and Neumann remainder terms. This, at least heuristically, explains why Theorem 1.5 holds true.

## Mixed Dirichlet–Neumann Boundary Conditions

As discussed in the introduction, the minimisation problem (2) for Dirichlet eigenvalues under perimeter constraint is well-posed but the minimisation problem (4) for Neumann eigenvalues under perimeter constraint is ill-posed. Here we consider a non-trivial minimisation problem for eigenvalues of the Laplacian under mixed Dirichlet–Neumann, so-called Zaremba, boundary conditions under perimeter constraint which is well-posed and has the same asymptotic behaviour as in Theorem 1.1.

To do this we define a subcollection $\mathcal {O}_{d,L}$ of $\mathcal {O}_{d}$ such that for each domain in the collection there is a canonical way of prescribing the mixed boundary conditions and the minimisation problem itself is well-posed. The definition of this subcollection is subtle and may appear somewhat odd at first but it allows us to obtain uniform lower bounds on the Zaremba eigenvalues and deduce the continuity of the Zaremba eigenvalues over $\mathcal {O}_{d,L}$. Without further ado, we give the definition of this subcollection below.

Let $\wp$ be the canonical projection $\mathbb {R}^{d} \rightarrow \mathbb {R}^{d-1}$ which omits the final coordinate. Throughout the rest of this paper, $\wp$ will denote this projection. Given $\Omega \in \mathcal {O}_{d}$, its image under $\wp$, denoted $\wp (\Omega )$, is a convex domain in $\mathbb {R}^{d-1}$. For each $x' \in \wp (\Omega )$ we can define two functions $h^{+},h^{-}:\wp (\Omega ) \rightarrow \mathbb {R}$ by

$$\begin{aligned} h^{+}(x') = \sup \lbrace y \in \mathbb {R}: (x',y) \in \Omega \rbrace , \quad h^{-}(x') = \inf \lbrace y \in \mathbb {R}: (x',y) \in \Omega \rbrace . \end{aligned}$$

We call $h^{+}$ and $h^{-}$ the upper and lower profiles of $\Omega$ and as functions they are concave and convex respectively. These functions are well defined as any line passing through a convex domain intersects the boundary precisely twice. Given $L>0$, we say that $\Omega$ is a convex *L*-Lip domain if $h^{+}$ and $h^{-}$ are both *L*-Lipschitz and agree on the boundary of $\wp (\Omega )$, denoted $\partial \wp (\Omega )$. We denote the collection of all convex *L*-Lip domains in $\mathbb {R}^{d}$ by $\mathcal {O}_{d,L}$. We define the upper boundary of $\Omega$ by $\Gamma ^{+}:= \Gamma ^{+}(\Omega ):= \lbrace (x',h^{+}(x')): x' \in \wp (\Omega ) \rbrace \subset \partial \Omega$ and define the lower boundary $\Gamma ^{-}:= \Gamma ^{-}(\Omega )$ analogously.

Let $\Omega \in \mathcal {O}_{d,L}$. We define the Zaremba Sobolev space $\mathcal {H}_{0,\Gamma ^{-}}^{1}(\Omega )$ as the completion of the space

$$\begin{aligned} C_{0,\Gamma ^{-}}^{\infty }(\Omega ) = \lbrace \left. \phi \right| _{\Omega } \in C^{\infty }(\Omega ): \phi \in C_{0}^{\infty }(\mathbb {R}^{d}), \, d(\textrm{supp}(\phi ), \Gamma ^{-}) > 0\rbrace \end{aligned}$$

in the Sobolev norm

$$\begin{aligned} \Vert u\Vert _{\mathcal {H}^{1}}:= \left( \int _{\Omega } |\nabla u|^{2} + \int _{\Omega }u^{2}\right) ^{1/2}. \end{aligned}$$

Then, in the usual way, we define the Zaremba Laplacian $-\Delta _{\Omega }^{Z}$ on $\mathcal {L}^{2}(\Omega )$ via the Friedrich’s extension with domain

$$\begin{aligned} \textrm{dom}(-\Delta _{\Omega }^{Z}) = \left\{ u \in \mathcal {H}_{0,\Gamma ^{-}}^{1}(\Omega ): \Delta u \in \mathcal {L}^{2}(\Omega ), \, \left. \partial _{n}u\right| _{\Gamma ^{+}} = 0 \right\} , \end{aligned}$$

where the conditions in the definition of $\textrm{dom}(-\Delta _{\Omega }^{Z})$ are understood in the distributional sense. The Zaremba Laplacian $-\Delta _{\Omega }^{Z}$ has the associated symmetric bilinear form

$$\begin{aligned} Q(u,v) = \int _{\Omega } \nabla u \cdot \nabla v \end{aligned}$$

with domain $\textrm{dom}(Q) = \mathcal {H}_{0,\Gamma ^{-}}^{1}(\Omega )$ and one can deduce that $-\Delta _{\Omega }^{Z}$ has a discrete collection of positive eigenvalues accumulating only at $+\infty$, which we denote

$$\begin{aligned} 0< \zeta _{1}(\Omega ) < \zeta _{2}(\Omega ) \le \zeta _{3}(\Omega ) \le \cdots \uparrow +\infty , \end{aligned}$$

that have the variational characterisation

22 $$\begin{aligned} \zeta _{k}(\Omega ) = \min _{\begin{array}{c} S \subseteq \mathcal {H}_{0,\Gamma ^{-}}^{1}(\Omega ) \\ \dim (S) = k \end{array}} \max _{\begin{array}{c} u \in S \\ u\ne 0 \end{array}} \frac{\int _{\Omega } |\nabla u|^{2}}{\int _{\Omega } u^{2}}. \end{aligned}$$

For our purposes we only need the definition of $\mathcal {H}_{0,\Gamma ^{-}}^{1}(\Omega )$ and the variational characterisation given in (22). For a fuller discussion on defining Zaremba eigenvalues we direct the reader to [31, §2] and [30, §3.1.3], and the references therein.

Now that $\mathcal {O}_{d,L}$ has been defined and we have defined Zaremba eigenvalues on domains lying in $\mathcal {O}_{d,L}$ we are ready to state our main results.

### Theorem 5.1

For any $d\ge 2$ and $L>0$, for all $k\ge 1$ there exists a minimiser $\Omega _{k}^{*}$ to the problem

23 $$\begin{aligned} \inf \lbrace \zeta _{k}(\Omega ): \Omega \in \mathcal {O}_{d,L}, \, |\partial \Omega | = 1\rbrace . \end{aligned}$$

Moreover, any sequence $\Omega _{k}^{*}$ of minimisers is non-degenerate, i.e. $\liminf _{k\rightarrow +\infty }|\Omega _{k}^{*}|>0$, and any accumulation point, up to possible rigid planar motions, of $\Omega _{k}^{*}$ is a solution to the isoperimetric problem over $\mathcal {O}_{d,L}$, which is necessarily symmetric, up to a translation, about the hyperplane $\lbrace x_{d} = 0\rbrace$.

As we shall soon argue, for any $k\ge 1$ and $d\ge 3$,

24 $$\begin{aligned} \inf \left\{ \zeta _{k}(\Omega ): \Omega \in \bigcup _{L>0} \mathcal {O}_{d,L}, \, |\partial \Omega | = 1\right\} = 0 \end{aligned}$$

and so without a uniform *L*-Lipschitz constraint the conclusion of Theorem 5.1 fails to hold. Moreover, for any $k\ge 1$ and $d\ge 3$,

25 $$\begin{aligned} \inf \left\{ \mu _{k}(\Omega ): \Omega \in \mathcal {O}_{d,L}, \, |\partial \Omega | = 1\right\} = 0 \end{aligned}$$

for all $k\in \mathbb {N}$ and so the Zaremba eigenvalues behave fundamentally differently to Neumann eigenvalues over the collection $\mathcal {O}_{d,L}$.

We now briefly illustrate (24) and (25) as an example when $d=3$, the higher dimensional cases can be done similarly.

### Example 5.2

Let $0< \varepsilon < 1$ and set $R_{\varepsilon } = (0,\varepsilon ) \times (0,\varepsilon )$. Define $f_{L,\varepsilon }: R_{\varepsilon } \rightarrow \mathbb {R}$ by $f_{L,\varepsilon }(x,y) = \min \lbrace L d((x,y), \partial R_{\varepsilon }), \varepsilon ^{-1}\rbrace$ and let

$$\begin{aligned} \Omega _{L,\varepsilon }:= \lbrace (x,y,z): (x,y)\in R_{\varepsilon }, \, 0< z < f_{L,\varepsilon }(x,y) \rbrace , \end{aligned}$$

which lies in $\mathcal {O}_{3,L}$. Let $u_{j}(x,y,z)= \sin (\pi (j+1/2) \varepsilon z)$ for $1 \le j \le k$ and let $V = \textrm{span}\lbrace u_{1},\ldots ,u_{k} \rbrace$. Note that the collection $\lbrace u_{1},\ldots ,u_{k} \rbrace$ is a linearly independent set and so *V* can be used as a test space in the variational characterisation of the *k*-th Zaremba eigenvalue for $\Omega _{L,\varepsilon }$, see (22). Then we see that

$$\begin{aligned} \begin{aligned} \zeta _{k}(\Omega _{L,\varepsilon }) \le \max _{0\ne v \in V} \frac{\displaystyle \int _{\Omega _{L,\varepsilon }} \left| \nabla v \right| ^{2}}{\displaystyle \int _{\Omega _{L,\varepsilon }} \left| v \right| ^{2}} = \max _{0\ne v \in V} \frac{\displaystyle \int _{\mathbb {R}^{3}} \left| \nabla v \right| ^{2}\mathbbm {1}_{\Omega _{L,\varepsilon }}}{\displaystyle \int _{\mathbb {R}^{3}} \left| v \right| ^{2}\mathbbm {1}_{\Omega _{L,\varepsilon }}}. \end{aligned} \end{aligned}$$

Noting that $\mathbbm {1}_{\Omega _{L,\varepsilon }} \rightarrow \mathbbm {1}_{R_{\varepsilon } \times (0,\varepsilon ^{-1})}$ in $\mathcal {L}^{p}(\mathbb {R}^{3})$ for any $p\in [1,+\infty )$, we see that

$$\begin{aligned} \max _{0\ne v \in V} \frac{\displaystyle \int _{\mathbb {R}^{3}} \left| \nabla v \right| ^{2}\mathbbm {1}_{\Omega _{L,\varepsilon }}}{\displaystyle \int _{\mathbb {R}^{3}} \left| v \right| ^{2}\mathbbm {1}_{\Omega _{L,\varepsilon }}} \rightarrow \max _{0\ne v \in V} \frac{\displaystyle \int _{R_{\varepsilon }\times (0,\varepsilon ^{-1})} \left| \nabla v \right| ^{2}}{\displaystyle \int _{R_{\varepsilon }\times (0,\varepsilon ^{-1})} \left| v \right| ^{2}} =\pi ^{2}(k+1/2)^{2}\varepsilon ^{2} \end{aligned}$$

as $L\rightarrow +\infty$. Moreover, $|\partial \Omega _{L,\varepsilon }| \rightarrow 4+2\varepsilon ^{2}$ as $L\rightarrow +\infty$. Since $0< \varepsilon < 1$ was arbitrary, by the properties of Zaremba eigenvalues under homothety, i.e. $\zeta _{k}(s\Omega ) = s^{-2}\zeta _{k}(\Omega )$ for any $s>0$, we see that (24) indeed holds for any $k\ge 1$ when $d=3$. Note that $\mathcal {O}_{d,L}$ is closed under homothety here.

Now set $S_{\varepsilon }:= (0,\varepsilon ^{-1})\times (0,\varepsilon )$ and set $g_{L,\varepsilon }: S_{\varepsilon } \rightarrow (0,+\infty )$ by $g_{L,\varepsilon }(x,y):= L d((x,y),\partial S_{\varepsilon })$ and define the domain $D_{\varepsilon }$ by

$$\begin{aligned} D_{\varepsilon }:= \lbrace (x,y,z): (x,y) \in S_{\varepsilon }, \, -g_{L,\varepsilon }(x,y)< z < g_{L,\varepsilon }(x,y) \rbrace . \end{aligned}$$

We have that $D_{\varepsilon } \in \mathcal {O}_{3,L}$ and that $|\partial D_{\varepsilon }| = 2\sqrt{1+L^{2}}$ for all $0< \varepsilon < 1$. Letting $u_{j}(x,y,z) = \cos (\pi j \varepsilon x)$ for $1\le j \le k$, one sees that the collection $\lbrace u_{1},\ldots ,u_{k} \rbrace$ is a linearly independent set. Denoting $V = \textrm{span}\lbrace u_{1},\ldots ,u_{k} \rbrace$, we can use *V* as a test space in the variational characterisation of the *k*-th Neumann eigenvalue for $D_{\varepsilon }$. Doing this, where we $\mathcal {L}^{2}$-normalise functions in *V* by assumption to remove the denominator, we obtain

$$\begin{aligned} \mu _{k}(D_{\varepsilon })\le &   \max _{\begin{array}{c} v = \alpha _{1}u_{1}+\cdots +\alpha _{k}u_{k} \in V \\ \Vert v\Vert _{\mathcal {L}^{2}(D_{\varepsilon })} = 1 \end{array}} \int _{D_{\varepsilon }} |\nabla v|^{2} \\= &   \max _{\begin{array}{c} v = \alpha _{1}u_{1}+\cdots +\alpha _{k}u_{k}\in V \\ \Vert v\Vert _{\mathcal {L}^{2}(D_{\varepsilon })} = 1 \end{array}} \int _{0}^{\varepsilon ^{-1}} dx \, \int _{0}^{\varepsilon } dy \, \int _{-g_{\varepsilon }(x,y)}^{g_{\varepsilon }(x,y)} dz\, \left| \nabla \sum _{j} \alpha _{j} u_{j}(x,y,z)\right| ^{2} \\\le &   L\varepsilon ^{2} \max _{\begin{array}{c} v = \alpha _{1}u_{1}+\cdots +\alpha _{k}u_{k}\in V\\ \Vert v\Vert _{\mathcal {L}^{2}(D_{\varepsilon })} = 1 \end{array}} \int _{0}^{\varepsilon ^{-1}} dx \, \left| \nabla \sum _{j} \alpha _{j} \cos (\pi j \varepsilon x)\right| ^{2} \\= &   L\pi ^{2}k^{2} \varepsilon ^{4}. \end{aligned}$$

Since $0< \varepsilon < 1$ was arbitrary, by the scaling properties of Neumann eigenvalues under homothety, i.e. $\mu _{k}(s\Omega ) = s^{-2}\mu _{k}(\Omega )$ for any $s>0$, we see that (25) indeed holds for any $k\ge 1$ when $d=3$. Again, note that $\mathcal {O}_{d,L}$ is closed under homothety here.

In the same way as one proves Theorem 5.1, one can also deduce the analogous result in the case of diameter constraint.

### Theorem 5.3

For any $d\ge 2$ and $L>0$, for all $k\ge 1$ there exists a minimiser $\Omega _{k}^{*}$ to the problem

$$\begin{aligned} \inf \lbrace \zeta _{k}(\Omega ): \Omega \in \mathcal {O}_{d,L}, \, \textrm{diam}(\Omega ) = 1\rbrace . \end{aligned}$$

Moreover, any sequence $\Omega _{k}^{*}$ of minimisers is non-degenerate and any accumulation point, up to possible rigid planar motions, of $\Omega _{k}^{*}$ is a solution to the isodiametric problem over $\mathcal {O}_{d,L}$.

To make our results clearer, let us illuminate Theorem 5.1 through an example in two dimensions.

### Example 5.4

**Fig. 1:**
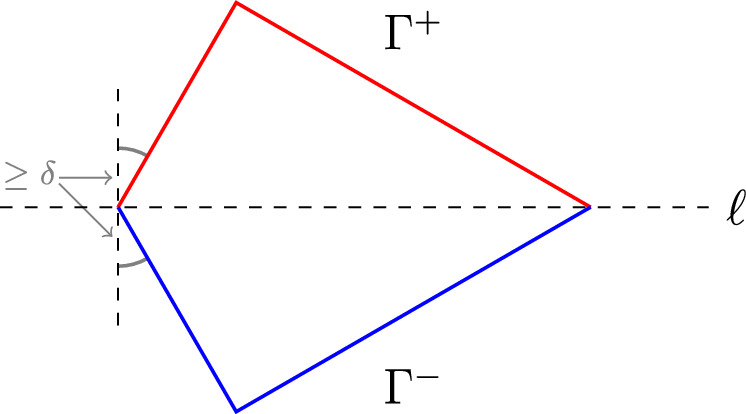
An example of symmetric Zaremba boundary conditions on a kite about its axis of symmetry, with Dirichlet boundary conditions denoted in blue and Neumann boundary conditions denoted in red (Color figure online)

Fix $0< \delta \le \frac{\pi }{4}$. Let $\Omega \subset \mathbb {R}^{2}$ be a kite of unit perimeter, let $\ell$ be the line of symmetry of $\Omega$ and assume that the angles that $\ell$ passes through are less than or equal to $\pi -2\delta$, see Fig. 1 for an example of this. The collection of such kites is closed in the Hausdorff metric. Partition the boundary of the kite into two disjoint relatively open components $\Gamma ^{+}$ and $\Gamma ^{-}$ which lie on either side of $\ell$ and, up to a set of measure zero, cover $\partial \Omega$. Then one can define the Zaremba Laplacian for kites in the way described earlier in this subsection. Then, arguing as in the proof of Theorem 5.1, this gives that for $k\ge 1$ there exists a minimiser $\Omega _{k}^{*}$ of the *k*-th Zaremba eigenvalue among such kites with unit perimeter, and the isoperimetric problem for kites implies that any sequence of such optimisers must converge to the square of unit perimeter as $k\rightarrow +\infty$.

As a corollary, one can carry out the same for rhombii where $\ell$ is the line of symmetry passing through the smallest opposite pair of interior angles. Then under perimeter constraint, again one has existence of optimisers for $k\ge 1$ and that the optimisers necessarily converge to the square of unit perimeter as $k\rightarrow + \infty$.

We now turn our attention to proving Theorems 5.1 and 5.3. An easy but key observation to make is that $\mathcal {O}_{d,L}$ is closed under homothety. We begin the section by showing $\mathcal {O}_{d,L}$ is closed in the Hausdorff topology provided that one does not have degeneracy of the volume in the limit. Then we use the definition of $\mathcal {O}_{d,L}$ to prove the continuity of these Zaremba eigenvalues in the Hausdorff topology and then prove a Li-Yau type lower bound for these eigenvalues. Both the proofs of the continuity and the lower bound require the use of Sobolev extension operators and the choice of definition of $\mathcal {O}_{d,L}$ will become more apparent throughout this section.

### Properties of $\mathcal {O}_{d,L}$

#### Lemma 5.5

If $\Omega _{n} \in \mathcal {O}_{d,L}$ is a sequence of domains Hausdorff converging to $\Omega \in \mathcal {O}_{d}$ as $n\rightarrow +\infty$, then $\Omega \in \mathcal {O}_{d,L}$.

#### Proof

By the invariance of $\mathcal {O}_{d,L}$ under homothety and translations and standard properties of Hausdorff convergence of convex domains, it suffices to prove the result in the case of sequences $\Omega _{n}$ that lie in $\mathcal {O}_{d,L}$ which Hausdorff converge to some bounded convex domain $\Omega$ as $n\rightarrow +\infty$ and for which $\Omega _{n} \subset \Omega$ for each *n*. Let $h_{n}^{+}: \wp (\Omega _{n}) \rightarrow \mathbb {R}$ be the upper height function of $\Omega _{n}$ and $h^{+}$ the upper height function of $\Omega$. Now fix $x',y'\in \wp (\Omega )$ and let $\varepsilon = \frac{1}{2}\min \lbrace d(x',\partial \wp (\Omega )),d(y',\partial \wp (\Omega ))\rbrace$. Then it is easy to see that $\wp (\Omega _{n})$ Hausdorff converges to $\wp (\Omega )$ as $n\rightarrow +\infty$ so we have that $B(x',\varepsilon ),B(y',\varepsilon )\subset \wp (\Omega _{n})$ for *n* sufficiently large. Now also for *n* sufficiently large we see that $d(\partial \Omega , \partial \Omega _{n}) < \varepsilon$ by standard results of Hausdorff convergence of convex domains and so, as $\Omega _{n} \subset \Omega$, it is clear that there exist sequences $(x_{n}',h_{n}^{+}(x_{n}')),(y_{n}',h_{n}^{+}(y_{n}')) \in \partial \Omega _{n}$ converging to $(x',h^{+}(x')) (y',h^{+}(y'))\in \partial \Omega$ as $n\rightarrow +\infty$. In particular, we see that these sequences can be chosen so that

$$\begin{aligned} \Vert (x_{n}',h_{n}^{+}(x_{n}'))-(x',h^{+}(x'))\Vert _{2}, \Vert (y_{n}',h_{n}^{+}(y_{n}'))-(y',h^{+}(y'))\Vert _{2} \le d_{H}(\partial \Omega _{n},\partial \Omega ). \end{aligned}$$

Then

$$\begin{aligned} \begin{aligned} |h^{+}(x')-h^{+}(y')|&\le |h^{+}(x')-h_{n}^{+}(x_{n}')| + |h_{n}^{+}(x_{n}')-h_{n}^{+}(y_{n}')| + |h_{n}^{+}(y_{n}')-h^{+}(y')| \\&\le 2d_{H}(\partial \Omega _{n},\partial \Omega ) + L|x_{n}'-y_{n}'|. \end{aligned} \end{aligned}$$

Taking the limit as $n\rightarrow +\infty$ we see that $h^{+}$ is *L*-Lipschitz. Similarly one can show that $h^{-}$, the lower height function of $\Omega$, is *L*-Lipschitz. The fact that $h^{+}$ and $h^{-}$ agree on the boundary $\partial \wp (\Omega )$ is easy to argue by contradiction. $\square$

#### Lemma 5.6

If $\Omega _{n}$ is a sequence of domains in $\mathcal {O}_{d,L}$ Hausdorff converging to a domain $\Omega \in \mathcal {O}_{d,L}$ as $n\rightarrow +\infty$, then $\Gamma _{n}^{-}:=\Gamma ^{-}(\Omega _{n})$ Hausdorff converges to $\Gamma ^{-}:=\Gamma ^{-}(\Omega )$ as $n\rightarrow +\infty$.

#### Proof

As in the proof of Lemma 5.5, we may assume that $\Omega _{n} \subset \Omega$ for each $n\in \mathbb {N}$. For $\delta > 0$ define the compact subset

$$\begin{aligned} K_{\delta }:= \lbrace (x',y) \in \wp (\Omega )\times \mathbb {R}: h^{-}(x') + \delta \le y \le h^{+}(x') - \delta \rbrace \subset \Omega . \end{aligned}$$

Then for *n* sufficiently large, we see that $K_{\delta } \subset \Omega _{n}$. Fix $(x',h^{-}(x'))\in \Gamma ^{-}(\Omega )$, then let $x_{\delta }'$ be the closest point in $\wp (K_{\delta })$ to $x'$. Then clearly $|x'-x_{\delta }'| \le d_{H}(K_{\delta },\Omega )$ and so

$$\begin{aligned} \begin{aligned} |h^{-}(x')-h_{n}^{-}(x_{\delta }')|&\le |h^{-}(x')-h^{-}(x_{\delta }')| + |h^{-}(x_{\delta }')-h_{n}^{-}(x_{\delta }')| \\&\le Ld_{H}(K_{\delta },\Omega ) + \delta . \end{aligned} \end{aligned}$$

Since $\delta >0$ was arbitrary we see that $\sup _{x\in \Gamma ^{-}(\Omega )}\inf _{y\in \Gamma ^{-}(\Omega _{n})} \Vert x-y\Vert _{2} \rightarrow 0$ as $n\rightarrow +\infty$. One can then deduce that $\sup _{x\in \Gamma ^{-}(\Omega _{n})}\inf _{y\in \Gamma ^{-}(\Omega )} \Vert x-y\Vert _{2} \rightarrow 0$ as $n\rightarrow +\infty$ similarly. $\square$

### Continuity of the $\zeta _{k}$

We now move on to prove the continuity of these Zaremba eigenvalues in the Hausdorff topology. In [14], Chenais proved the continuity of solutions to the Neumann problem for domains satisfying a uniform cone condition with respect to the Hausdorff metric. A crucial part of Chenais’ proof is to show that over such a collection of domains there exists a uniform constant such that there exists a Sobolev extension operator $\mathcal {H}^{1}(\Omega ) \rightarrow \mathcal {H}^{1}(\mathbb {R}^{d})$ whose norm is at most this uniform constant. Then from the continuity of the solutions to the Neumann problem, one can prove the continuity of Neumann eigenvalues with respect to the Hausdorff metric, see [25, §3]. The issue that arises in the Zaremba problem is that one wants to extend by zero on the Dirichlet parts of the boundary and extend non-trivially along the Neumann parts of the boundary. This is an inherently tricky situation as you wish to extend by zero near/on Dirichlet parts of the boundary but cannot do so on the Neumann parts of the boundary. Our definition of $\mathcal {O}_{d,L}$ allows us to define an extension operator which for any $\Omega \in \mathcal {O}_{d,L}$ extends any $u \in H_{0,\Gamma ^{-}}^{1}(\Omega )$ by zero below $\Gamma ^{-}$ and ‘into $\mathcal {H}^{1}$ above $\Gamma ^{+}$’. Moreover, we can uniformly bound such operators over $\mathcal {O}_{d,L}$. For a precise formulation of this see Corollary 5.8. Then by similar arguments to Chenais, we prove the continuity of Zaremba eigenvalues over the collection.

#### Lemma 5.7

[19, Lemma 2.91] There exists a constant $C_{L} > 0$ depending only on $L>0$ such that for any *L*-Lipschitz function $f: \mathbb {R}^{d-1} \rightarrow \mathbb {R}$, there exists a Sobolev extension operator $\mathcal {E}: \mathcal {H}^{1}(\Omega _{f}) \rightarrow \mathcal {H}^{1}(\mathbb {R}^{d})$, where

26 $$\begin{aligned} \Omega _{f}:= \lbrace (x',y) \in \mathbb {R}^{d-1} \times \mathbb {R}: y < f(x') \rbrace , \end{aligned}$$

with $\Vert \mathcal {E}[u] \Vert _{\mathcal {L}^{2}(\mathbb {R}^{d}\backslash \Omega _{f})} \le \sqrt{2}\Vert u \Vert _{\mathcal {L}^{2}(\Omega _{f})}$ and $\Vert \nabla \mathcal {E}[u] \Vert _{\mathcal {L}^{2}(\mathbb {R}^{d}\backslash \Omega _{f})} \le C_{L}\Vert \nabla u \Vert _{\mathcal {L}^{2}(\Omega _{f})}$ for any $u\in \mathcal {H}^{1}(\Omega _{f})$. Explicitly, we have that

27 $$\begin{aligned} \mathcal {E}[u](x',y) = {\left\{ \begin{array}{ll} u(x',y), &  y < f(x'), \\ u(x',-y+2f(x')), &  y > f(x'). \end{array}\right. } \end{aligned}$$

A detailed analysis of this Sobolev extension operator is not necessary for our means, the only important point for us here is the following immediate corollary.

#### Corollary 5.8

There exists a constant $C_{L} > 0$ depending only on $L>0$ such that for any $\Omega \in \mathcal {O}_{d,L}$ there exists an extension operator $\mathcal {E}_{\Omega }:\mathcal {H}_{0,\Gamma ^{-}}^{1}(\Omega ) \rightarrow \mathcal {H}_{0}^{1}(\Omega _{\infty })$, where

$$\begin{aligned} \Omega _{\infty } = \lbrace (x',y) \in \wp (\Omega ) \times \mathbb {R}: y > h_{-}(x')\rbrace , \end{aligned}$$

with $\Vert \mathcal {E}_{\Omega }[u] \Vert _{\mathcal {L}^{2}(\mathbb {R}^{d}\backslash \Omega )} \le \sqrt{2}\Vert u \Vert _{\mathcal {L}^{2}(\Omega )}$ and $\Vert \nabla \mathcal {E}_{\Omega }[u] \Vert _{\mathcal {L}^{2}(\mathbb {R}^{d}\backslash \Omega )} \le C_{L}\Vert u \Vert _{\mathcal {L}^{2}(\Omega )}$ for any $u\in \mathcal {H}_{0,\Gamma ^{-}}^{1}(\Omega )$.

#### Proof

Take any $\phi \in C_{0,\Gamma ^{-}}^{\infty }(\Omega ) \cap C^{\infty }(\overline{\Omega })$. By a theorem of McShane in [35], we can extend $h^{+}:\wp (\Omega ) \rightarrow \mathbb {R}$ to an *L*-Lipschitz function $\widetilde{h}^{+}: \mathbb {R}^{d-1} \rightarrow \mathbb {R}$. Defining $\Omega _{\widetilde{h}^{+}}$ as in (26), by extending by zero $\phi \in \mathcal {H}^{1}(\Omega _{\widetilde{h}_{+}})$, and by the definition of $\mathcal {E}$ in (27) it is clear that one must have $\mathcal {E}[\phi ] \in \mathcal {H}_{0}^{1}(\Omega _{\infty })$. Define $\mathcal {E}_{\Omega }[\phi ]$ in this way. Then by the density of $C_{0,\Gamma ^{-}}^{\infty }(\Omega )\cap C^{\infty }(\overline{\Omega })$ in $\mathcal {H}_{0,\Gamma ^{-}}^{1}(\Omega )$, the result immediately follows. $\square$

#### Lemma 5.9

For each $k\in \mathbb {N}$, if $\Omega _{n}\in \mathcal {O}_{d,L}$ Hausdorff converges to $\Omega \in \mathcal {O}_{d,L}$ as $n\rightarrow +\infty$ then $\zeta _{k}(\Omega _{n}) \rightarrow \zeta _{k}(\Omega )$ as $n\rightarrow +\infty$.

#### Proof

Since we know that $\Omega _{n}$ Hausdorff converges to $\Omega$, we know that there exists $\beta _{n} \rightarrow 1$ such that $\beta _{n}\Omega _{n} \subseteq \Omega$, up to a possible translation, for *n* sufficiently large. From here onwards, we follow the ideas of the proof of Proposition IV.1 in [14]. Fix $f\in \mathcal {L}^{2}(\Omega )$. By the Riesz–Fréchet representation theorem there exists a unique $u_{n} \in \mathcal {H}_{0,\Gamma ^{-}}^{1}(\beta _{n}\Omega _{n})$ such that

$$\begin{aligned} \int _{\Omega }\mathbbm {1}_{\beta _{n}\Omega _{n}}\nabla u_{n} \cdot \nabla \phi + \int _{\Omega }\mathbbm {1}_{\beta _{n}\Omega _{n}}u_{n}\phi = \int _{\Omega } \mathbbm {1}_{\beta _{n}\Omega _{n}} f\phi , \quad \forall \phi \in C_{0,\Gamma ^{-}}^{\infty }(\beta _{n}\Omega _{n}) \end{aligned}$$

with $\Vert u_{n} \Vert _{\mathcal {H}^{1}(\beta _{n}\Omega _{n})} = \Vert f\Vert _{\mathcal {L}^{2}(\beta _{n}\Omega _{n})} \le \Vert f\Vert _{\mathcal {L}^{2}(\Omega )}.$ Then we see that we can extend each $u_{n}\in \mathcal {H}_{0,\Gamma ^{-}}^{1}(\beta _{n}\Omega _{n})$ via $\mathcal {E}_{\Omega _{n}}$, as defined in Corollary 5.8, to a function $\bar{u}_{n} \in \mathcal {H}_{0,\Gamma ^{-}}^{1}(\Omega )$ with $\Vert \bar{u}_{n} \Vert _{\mathcal {H}^{1}(\Omega )} \le C_{L}\Vert f\Vert _{\mathcal {L}^{2}(\Omega )}$. By the Banach–Alaoglu theorem, up to a subsequence, $\bar{u}_{n} \rightharpoonup u$ in $\mathcal {H}_{0,\Gamma }^{1}(\Omega )$ as $n\rightarrow +\infty$. We now show that *u* must be the unique solution to

28 $$\begin{aligned} \int _{\Omega }\nabla u \cdot \nabla \phi + \int _{\Omega }u\phi = \int _{\Omega } f\phi , \quad \forall \phi \in C_{0,\Gamma ^{-}}^{\infty }(\Omega ). \end{aligned}$$

Fix $\phi \in C_{0,\Gamma ^{-}}^{\infty }(\Omega )$. Then by Lemma 5.6, we see that the support of $\phi$ is at a positive distance from $\Gamma ^{-}(\Omega _{n})$ for *n* sufficiently large, and so $\left. \phi \right| _{\beta _{n}\Omega _{n}} \in \mathcal {H}_{0,\Gamma ^{-}}^{1}(\beta _{n}\Omega _{n})$ for *n* sufficiently large. Thus, for *n* sufficiently large

$$\begin{aligned} \int _{\Omega }\mathbbm {1}_{\beta _{n}\Omega _{n}}\nabla \bar{u}_{n} \cdot \nabla \phi + \int _{\Omega }\mathbbm {1}_{\beta _{n}\Omega _{n}}\bar{u}_{n}\phi = \int _{\Omega } \mathbbm {1}_{\beta _{n}\Omega _{n}} f\phi . \end{aligned}$$

Following the arguments in [14, Prop. IV.1], it is clear that if we take the limit $n\rightarrow +\infty$,

$$\begin{aligned} \int _{\Omega }\nabla u \cdot \nabla \phi + \int _{\Omega }u\phi = \int _{\Omega } f\phi . \end{aligned}$$

Since $\phi \in C_{0,\Gamma ^{-}}^{\infty }(\Omega )$ was arbitrary, *u* is indeed the solution to (28) as desired. Moreover, $\bar{u}_{n}\rightarrow u$ in $\mathcal {L}^{2}(\Omega )$ by the Rellich–Kondrachov compactness theorem since $\bar{u}_{n} \rightharpoonup u$ in $\mathcal {H}_{0,\Gamma ^{-}}^{1}(\Omega )$. Now following the proof of Theorem 2.3.2. in [25], we see that $\zeta _{k}(\beta _{n}\Omega _{n}) \rightarrow \zeta _{k}(\Omega )$. Then noting that $\zeta _{k}(\beta _{n}\Omega _{n}) = (\beta _{n})^{-2}\zeta _{k}(\Omega _{n})$ we obtain the result. $\square$

### Proof of Theorems 5.1 and 5.3

With the continuity of Zaremba eigenvalues over $\mathcal {O}_{d,L}$ in hand, we now prove the existence of minimisers using the extension operator from Corollary 5.8.

#### Lemma 5.10

For each $k\ge 1$ there exists a minimiser $\Omega _{k}^{*}$ to (23).

#### Proof

Let $\delta = \Vert h^{+}-h^{-} \Vert _{\infty }$. Then one sees that, up to a possible translation, $\Omega \subset \wp (\Omega ) \times (0,\delta )$. We can extend the first Zaremba eigenfunction of $\Omega$ to the Sobolev space $\mathcal {H}_{0,\wp (\Omega )\times \lbrace 0\rbrace }^{1}(\wp (\Omega ) \times (0,\delta ))$. Hence, from the variational characterisation of the first Zaremba eigenvalue, we see that

$$\begin{aligned} \widetilde{\zeta }_{1}(\wp (\Omega ) \times (0,\delta )) \le C_{L} \zeta _{1}(\Omega ) \end{aligned}$$

where $\widetilde{\zeta }_{1}(\wp (\Omega ) \times (0,\delta ))$ is the first eigenvalue of the Zaremba Laplacian on $\wp (\Omega ) \times (0,\delta )$ with Dirichlet boundary conditions on $\wp (\Omega )\times \lbrace 0\rbrace$. By separation of variables one can deduce that

$$\begin{aligned} \widetilde{\zeta }_{1}(\wp (\Omega ) \times (0,\delta )) = \mu _{1}(\wp (\Omega )) + \frac{\pi ^{2}}{4\delta ^{2}} = \frac{\pi ^{2}}{4\delta ^{2}}. \end{aligned}$$

And so we see that

$$\begin{aligned} \zeta _{k}(\Omega ) \ge \zeta _{1}(\Omega ) \ge \frac{\pi ^{2}}{4C_{L} \delta ^{2}} \uparrow +\infty \end{aligned}$$

as $\delta \downarrow 0$. Hence, we must have that $\delta$ is uniformly bounded and so the inradii of the sets must be uniformly bounded from below. Let $\Omega _{n}$ be a minimising sequence for the infimum, then since the inradius is uniformly bounded from below we have that, up to a sequence of translations, there exists a Hausdorff convergent subsequence $\Omega _{n_{j}}$ converging to some domain $\Omega \in \mathcal {O}_{d,L}$ as $j\rightarrow +\infty$ by Lemmas 3.3 and 5.5. Since the Zaremba eigenvalues are continuous in this topology, see Lemma 5.9, $\zeta _{k}(\Omega _{n_{j}}) \rightarrow \zeta _{k}(\Omega )$ as $j\rightarrow +\infty$ and we are done. $\square$

We now give a lower bound for Zaremba eigenvalues in the spirit of the classical Li–Yau bound, see [32, Cor. 1], for Dirichlet eigenvalues.

#### Lemma 5.11

There exists a constant $C_{d,L} > 0$, depending only on $d\ge 2$ and $L>0$, such that for any $\varepsilon > 0$

$$\begin{aligned} \zeta _{k}(\Omega ) \ge \frac{C_{d,L}k^{2/d}}{(|\Omega | + \varepsilon |\wp (\Omega )|^{d/(d-1)})^{2/d}} - ((d-1)L^{2}+1)\frac{1}{\varepsilon ^{2}|\wp (\Omega )|^{2/(d-1)}} \end{aligned}$$

for all $$\Omega \in \mathcal {O}_{d,L}$$.

#### Proof

Let $\varepsilon > 0$. Fix $\Omega \in \mathcal {O}_{d,L}$ and define the set

$$\begin{aligned} \Omega ^{\varepsilon } = \lbrace (x',y) \in \wp (\Omega ) \times \mathbb {R}: h^{-}(x')< y < h^{+}(x') + \varepsilon \rbrace . \end{aligned}$$

Further for $\varepsilon >0$, define the function $\chi _{\varepsilon }: \wp (\Omega ) \times \mathbb {R} \rightarrow [0,1]$ by

$$\begin{aligned} \chi _{\varepsilon }(x',y):= {\left\{ \begin{array}{ll} 1, &  y \le h^{+}(x'), \\ 1-\frac{(y-h^{+}(x'))}{\varepsilon }, &  h^{+}(x')< y < h^{+}(x') +\varepsilon , \\ 0 &  y \ge h^{+}(x') + \varepsilon \end{array}\right. } \end{aligned}$$

Let $\mathcal {E}$ be the Sobolev extension operator given in Corollary 5.8. For any $u\in \mathcal {H}_{0,\Gamma ^{-}}^{1}(\Omega )$, we have that $\chi _{\varepsilon }\mathcal {E}[u] \in \mathcal {H}_{0}^{1}(\Omega ^{\varepsilon })$. Moreover, let $S_{k} = \lbrace u_{1},\ldots , u_{k}\rbrace$ denote the span of the first *k* orthonormal eigenfunctions of $-\Delta _{\Omega }^{Z}$. Then the collection $\lbrace \chi _{\varepsilon }\mathcal {E}[u_{1}],\ldots , \chi _{\varepsilon }\mathcal {E}[u_{k}]\rbrace \subset \mathcal {H}_{0}^{1}(\Omega ^{\varepsilon })$ is linearly independent and so we pass the span of these functions as a trial space into the variational formulation for the *k*-th Dirichlet eigenvalue of $\Omega ^{\varepsilon }$.

Before proceeding let us make some relevant observations. Namely that, for any $u \in \mathcal {H}_{0,\Gamma ^{-}}^{1}(\Omega )$: $\Vert \mathcal {E}[u]\Vert _{\mathcal {L}^{2}(\Omega ^{\varepsilon })} \ge \Vert u\Vert _{\mathcal {L}^{2}(\Omega )}$ since $\mathcal {E}[u] \equiv u$ in $\Omega$; $\Vert \mathcal {E}[u]\Vert _{\mathcal {L}^{2}(\mathbb {R}^{d}\backslash \Omega )} \le \sqrt{2}\Vert u\Vert _{\mathcal {L}^{2}(\Omega )}$; and, $\Vert \nabla \mathcal {E}[u]\Vert _{\mathcal {L}^{2}(\mathbb {R}^{d}\backslash \Omega )} \le C_{L}\Vert \nabla u\Vert _{\mathcal {L}^{2}(\Omega )}$ as stated in Corollary 5.8.

By repeated use of the uniform bounds given in Corollary 5.8 and removing the denominator from the variational characterisation of the *k*-th Dirichlet eigenvalue of $\Omega ^{\varepsilon }$ by $\mathcal {L}^{2}$-normalising functions in $S_{k}$ in the definition of the maximum, we have that

$$\begin{aligned} \begin{aligned} \lambda _{k}(\Omega ^{\varepsilon })&\le \max _{\begin{array}{c} u\in S_{k} \\ \Vert u\Vert _{\mathcal {L}^{2}(\Omega )}=1 \end{array}} \int _{\Omega ^{\varepsilon }} \left| \nabla \left( \chi _{\varepsilon }\mathcal {E}[u]\right) \right| ^{2} \\&\le \max _{\begin{array}{c} u\in S_{k} \\ \Vert u\Vert _{\mathcal {L}^{2}(\Omega )}=1 \end{array}} \left\{ \int _{\Omega } \left| \nabla u\right| ^{2} + \int _{\Omega ^{\varepsilon }\backslash \Omega } \left| \chi _{\varepsilon }\nabla \mathcal {E}[u]+\mathcal {E}[u]\nabla \chi _{\varepsilon } \right| ^{2}\right\} \\&\le \max _{\begin{array}{c} u\in S_{k} \\ \Vert u\Vert _{\mathcal {L}^{2}(\Omega )}=1 \end{array}} \left\{ \int _{\Omega } \left| \nabla u\right| ^{2} + 2\int _{\Omega ^{\varepsilon }\backslash \Omega } \left| \chi _{\varepsilon }\nabla \mathcal {E}[u]\right| ^{2} + 2\int _{\Omega ^{\varepsilon }\backslash \Omega } \left| \mathcal {E}[u]\nabla \chi _{\varepsilon } \right| ^{2}\right\} \\&\le \max _{\begin{array}{c} u\in S_{k} \\ \Vert u\Vert _{\mathcal {L}^{2}(\Omega )}=1 \end{array}} \left\{ \int _{\Omega } \left| \nabla u\right| ^{2} + 2\int _{\mathbb {R}^{d}\backslash \Omega } \left| \nabla \mathcal {E}[u]\right| ^{2} + 2((d-1)L^{2}+1)\varepsilon ^{-2}\int _{\mathbb {R}^{d}\backslash \Omega } \left| \mathcal {E}[u]\right| ^{2}\right\} \\&\le \max _{\begin{array}{c} u\in S_{k} \\ \Vert u\Vert _{\mathcal {L}^{2}(\Omega )}=1 \end{array}} \left\{ (1+2C_{L})\int _{\Omega } \left| \nabla u\right| ^{2} + 2\sqrt{2}((d-1)L^{2}+1)\varepsilon ^{-2} \right\} \\&\le C_{L}' \left( \max _{\begin{array}{c} u\in S_{k} \\ \Vert u\Vert _{\mathcal {L}^{2}(\Omega )}=1 \end{array}} \left\{ \int _{\Omega } \left| \nabla u\right| ^{2} \right\} + ((d-1)L^{2}+1)\varepsilon ^{-2}\right) \\&= C_{L}' (\zeta _{k}(\Omega ) + ((d-1)L^{2}+1)\varepsilon ^{-2}). \end{aligned} \end{aligned}$$

By the classical Dirichlet eigenvalue lower bound of Li and Yau [32, Cor. 1], we see that

$$\begin{aligned} \lambda _{k}(\Omega ^{\varepsilon }) \ge \frac{dW_{d}k^{2/d}}{(d+2)|\Omega ^{\varepsilon }|^{2/d}}. \end{aligned}$$

Now, observing that $|\Omega ^{\varepsilon }|=|\Omega |+\varepsilon |\wp (\Omega )|$, we obtain that

$$\begin{aligned} \zeta _{k}(\Omega ) \ge \frac{C_{d,L}k^{2/d}}{(|\Omega |+\varepsilon |\wp (\Omega )|)^{2/d}} -((d-1) L^{2} +1) \varepsilon ^{-2}. \end{aligned}$$

Taking $\varepsilon = |\wp (\Omega )|^{1/(d-1)}\varepsilon '$ for some $\varepsilon '>0$, the result immediately follows. $\square$

Before proving Theorem 5.1, we now briefly look at the isoperimetric problem

29 $$\begin{aligned} \sup \left\{ |\Omega |: \Omega \in \mathcal {O}_{d,L}, \, |\partial \Omega | = 1 \right\} \end{aligned}$$

for domains in $\mathcal {O}_{d,L}$. It is clear that there exists a solution to the isoperimetric problem over $\mathcal {O}_{d,L}$, however we cannot say too much immediately as balls do not lie in $\mathcal {O}_{d,L}$. We now give some remarks on properties of solutions to (29).

By the results of Fuglede in [21], for $L >0$ large one can note that any solution to the isoperimetric problem must be (quantifiably) close to the ball of the same perimeter. Moreover, for any $\Omega \in \mathcal {O}_{d,L}$, its Steiner symmetrisation $\Omega ^{\#}$ about the hyperplane $\lbrace x_{d} = 0\rbrace$ defined by

$$\begin{aligned} \Omega ^{\#}:= \left\{ (x',y) \in \wp (\Omega ) \times \mathbb {R}: -h(x')< y < h(x')\right\} , \end{aligned}$$

where $h(x'):= (h^{+}(x')-h^{-}(x'))/2$, also lies in $\mathcal {O}_{d,L}$. Thus, we have that $|\Omega ^{\#}|=|\Omega |$ and $|\partial \Omega ^{\#}| \le |\partial \Omega |$, with equality if and only if $\Omega ^{\#}$ and $\Omega$ are isometric. Hence, any solution to the isoperimetric problem over $\mathcal {O}_{d,L}$ is necessarily symmetric about the hyperplane $\lbrace x_{d} = 0\rbrace$.

**Fig. 2:**
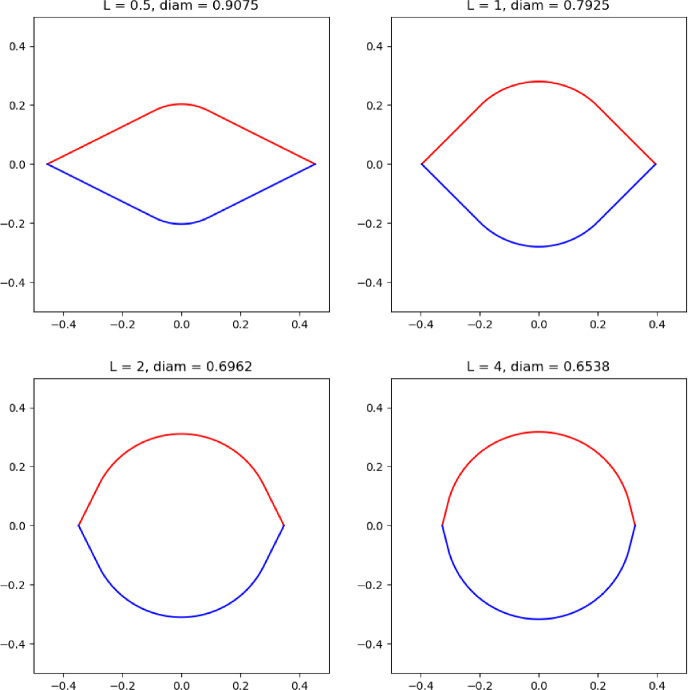
Numerically computed optimal solutions to the isoperimetric problem with unit perimeter over $\mathcal {O}_{2,L}$ with $L=0.5$ (top left), $L=1$ (top right), $L=2$ (bottom left) and $L=4$ (bottom right) (Color figure online)

As far as the author is aware, it is not known whether the solution to the isoperimetric problem over $\mathcal {O}_{d,L}$ is unique. In dimension two, it appears to be unique and the author has numerically computed solutions to the isoperimetric problem for $\mathcal {O}_{2,L}$, see Fig. 2.

We now show for any $\Omega \in \mathcal {O}_{d,L}$ that imposing the condition $|\partial \Omega |=1$ imposes constraints on $|\wp (\Omega )|$, which is the final ingredient needed to prove Theorem 5.1.

#### Lemma 5.12

Fix $\Omega \in \mathcal {O}_{d,L}$ and suppose that $|\partial \Omega | = 1$, then $\frac{1}{2\sqrt{L^{2}+1}}\le |\wp (\Omega )|\le \frac{1}{2}$.

#### Proof

For $\Omega \in \mathcal {O}_{d,L}$ observe that

$$\begin{aligned} 2 |\wp (\Omega )|\sqrt{1+L^{2}} \ge |\partial \Omega | = \int _{\wp (\Omega )} \sqrt{1+|\nabla h^{+}|^{2}} + \sqrt{1+|\nabla h^{-}|^{2}} \ge 2|\wp (\Omega )| \end{aligned}$$

and the result immediately follows. $\square$

#### Proof of Theorem 5.1

With our previous results in hand, we now follow the outline of the proof of Theorem 1.1 in [6] to prove Theorem 5.1. We already know the existence of minimisers to (23) from Lemma 5.10. Let $\Omega _{k}^{*}$ be any sequence of minimisers to (23) and let $\Omega ' \in \mathcal {O}_{d,L}$ with $|\partial \Omega '| =1$ be fixed. Using Lemmas 5.11 and 5.12, taking $\varepsilon >0$, we see that

$$\begin{aligned}\begin{aligned} \zeta _{k}(\Omega )&\ge \frac{C_{d,L}k^{2/d}}{(|\Omega | + \varepsilon |\wp (\Omega )|^{d/(d-1)})^{2/d}} - ((d-1)L^{2}+1)\frac{1}{\varepsilon ^{2}|\wp (\Omega )|^{2/(d-1)}} \\&\ge \frac{C_{d,L}k^{2/d}}{(|\Omega | + \varepsilon |\wp (\Omega )|^{d/(d-1)})^{2/d}} - ((d-1)L^{2}+1)\frac{(4+4L^{2})^{1/(d-1)}}{\varepsilon ^{2}} \end{aligned} \end{aligned}$$

for any $\Omega \in \mathcal {O}_{d,L}$ with $|\partial \Omega |=1$. Then observe that

$$\begin{aligned} \begin{aligned}&\frac{C_{d,L}k^{2/d}}{(|\Omega _{k}^{*}| + \varepsilon |\wp (\Omega _{k}^{*})|^{d/(d-1)})^{2/d}} - ((d-1)L^{2}+1)\frac{(4+4L^{2})^{1/(d-1)}}{\varepsilon ^{2}} \le \zeta _{k}(\Omega _{k}^{*}) \le \zeta _{k}(\Omega ') \\  &= \frac{W_{d}k^{2/d}}{|\Omega '|^{2/d}} + o(k^{2/d}) \end{aligned} \end{aligned}$$

and dividing through by $k^{2/d}$ and taking the limsup, we have, using Lemma 5.12 again,

$$\begin{aligned} \liminf _{k\rightarrow +\infty }|\Omega _{k}^{*}| + \frac{\varepsilon }{2}\ge \liminf _{k\rightarrow +\infty }\left[ |\Omega _{k}^{*}|^{2/d} +\varepsilon |\wp (\Omega _{k}^{*})|^{d/(d-1)}\right] \ge C_{d,L}(W_{d})^{-1}|\Omega |^{2/d} > 0. \end{aligned}$$

As $\varepsilon > 0$ was arbitrary, we see that the sequence of minimisers is non-degenerate. Now the only moot point to cover is that any accumulation point of the sequence $\Omega _{k}^{*}$, possibly up to translations of elements of the sequence, is indeed a solution to the isoperimetric problem over $\mathcal {O}_{d,L}$. Knowing the non-degeneracy, by Lemmas 3.3, 5.5 and the inequalities on p. 146 of [5], up to a sequence of translations, the $\Omega _{k}^{*}$ lie inside a sequentially compact subcollection of $\mathcal {O}_{d,L}$. Hence, there is a convergent subsequence $\Omega _{k_{j}}^{*}$, up to translating elements of the sequence, converging to some $\Omega _{\infty } \in \mathcal {O}_{d,L}$ as $j\rightarrow +\infty$. By Theorem 4.2 we see that

$$\begin{aligned} \lim _{j\rightarrow +\infty } \frac{\zeta _{k_{j}}(\Omega _{k_{j}})}{(k_{j})^{2/d}} = \frac{W_{d}}{|\Omega _{\infty }|^{2/d}}, \end{aligned}$$

using Dirichlet–Neumann bracketing i.e. $\mu _{k}(\Omega ) \le \zeta _{k}(\Omega ) \le \lambda _{k}(\Omega )$ for $\Omega \in \mathcal {O}_{d,L}$. Now if $\Omega _{\infty }$ is not a solution to the isoperimetric problem then we see that this would violate the optimality of the sequence $\Omega _{k}^{*}$. Moreover, $\Omega _{\infty }$ is necessarily symmetric about the hyperplane $\lbrace x_{d}=0\rbrace$, up to a translation, by our previous discussion. $\square$

The proof of Theorem 5.3 follows entirely analogously to the proof of Theorem 5.1 by noting that the condition $\textrm{diam}(\Omega ) =1$ implies that $|\wp (\Omega )| \le 2^{-(d-1)} \omega _{d-1}$ via the $(d-1)$-dimensional isodiametric inequality.

## Data Availability

This work did not involve any underlying data.

## Footnotes

1 The author would like to thank one of the anonymous referees for observing that the proofs of Propositions 3.1 and 3.2 can also be done in this way.

## References

[1] Antunes, P.R.S., Freitas, P.: Optimal spectral rectangles and lattice ellipses. Proc. R. Soc. Lond. Ser. A Math. Phys. Eng. Sci. **469**(2150), 20120492, 15 (2013). 10.1098/rspa.2012.0492

[2] Antunes, P.R.S., Freitas, P., Kennedy, J.B.: Asymptotic behaviour and numerical approximation of optimal eigenvalues of the Robin Laplacian. ESAIM Control Optim. Calc. Var. **19**(2), 438–459 (2013). 10.1051/cocv/2012016

[3] van den Berg, M., Bucur, D., Gittins, K.: Maximising Neumann eigenvalues on rectangles. Bull. Lond. Math. Soc. **48**(5), 877–894 (2016). 10.1112/blms/bdw049

[4] Bebendorf, M.: A note on the Poincaré inequality for convex domains. Z. Anal. Anwend. **22**(4), 751–756 (2003). 10.4171/ZAA/1170

[5] van den Berg, M.: On the minimization of Dirichlet eigenvalues. Bull. Lond. Math. Soc. **47**(1), 143–155 (2015). 10.1112/blms/bdu106

[6] Bucur, D., Freitas, P.: Asymptotic behaviour of optimal spectral planar domains with fixed perimeter. J. Math. Phys. **54**(5), 053504, 6 (2013). 10.1063/1.4803140

[7] van den Berg, M., Gittins, K.: Minimizing Dirichlet eigenvalues on cuboids of unit measure. Mathematika **63**(2), 469–482 (2017). 10.1112/S0025579316000413

[8] Bogosel, B., Henrot, A., Lucardesi, I.: Minimization of the eigenvalues of the Dirichlet-Laplacian with a diameter constraint. SIAM J. Math. Anal. **50**(5), 5337–5361 (2018). 10.1137/17M1162147

[9] Bogosel, B., Henrot, A., Michetti, M.: Optimization of Neumann eigenvalues under convexity and geometric constraints. SIAM J. Math. Anal. **56**(6), 7327–7349 (2024). 10.1137/24M1641099

[10] van den Berg, M., Iversen, M.: On the minimization of Dirichlet eigenvalues of the Laplace operator. J. Geom. Anal. **23**(2), 660–676 (2013). 10.1007/s12220-011-9258-0

[11] Bonnesen, T.: Über das isoperimetrische Defizit ebener Figuren. Math. Ann. **91**(3–4), 252–268 (1924). 10.1007/BF01556082

[12] Cavallina, L., Funano, K., Henrot, A., Lemenant, A., Lucardesi, I., Sakaguchi, S.: Two extremum problems for Neumann eigenvalues (2023). arXiv:2312.13747

[13] Courant, R., Hilbert, D.: Methods of Mathematical Physics, vol. I. Interscience Publishers Inc, New York (1953)

[14] Chenais, D.: On the existence of a solution in a domain identification problem. J. Math. Anal. Appl. **52**(2), 189–219 (1975). 10.1016/0022-247X(75)90091-8

[15] Cito, S.: Existence and regularity of optimal convex shapes for functionals involving the Robin eigenvalues. J. Convex Anal. **26**(3), 925–943 (2019)

[16] Courant, R.: Beweis des Satzes, daßvon allen homogenen Membranen gegebenen Umfanges und gegebener Spannung die kreisförmige den tiefsten Grundton besitzt. Math. Z. **1**(2–3), 321–328 (1918). 10.1007/BF01203619

[17] Frank, R.L., Larson, S.: Two-term spectral asymptotics for the Dirichlet Laplacian in a Lipschitz domain. J. Reine Angew. Math. **766**, 195–228 (2020). 10.1515/crelle-2019-0019

[18] Frank, R.L., Larson, S.: Riesz means asymptotics for Dirichlet and Neumann Laplacians on Lipschitz domains (2024). arXiv:2407.11808

[19] Frank, R.L., Laptev, A., Weidl, T.: Schrödinger Operators: Eigenvalues and Lieb-Thirring Inequalities, Volume 200 of Cambridge Studies in Advanced Mathematics. Cambridge University Press, Cambridge (2023). 10.1017/9781009218436

[20] Freitas, P.: Asymptotic behaviour of extremal averages of Laplacian eigenvalues. J. Stat. Phys. **167**(6), 1511–1518 (2017). 10.1007/s10955-017-1789-8

[21] Fuglede, B.: Stability in the isoperimetric problem for convex or nearly spherical domains in . Trans. Am. Math. Soc. **314**(2), 619–638 (1989). 10.2307/2001401

[22] Gittins, K., Larson, S.: Asymptotic behaviour of cuboids optimising Laplacian eigenvalues. Integr. Equ. Oper. Theory **89**(4), 607–629 (2017). 10.1007/s00020-017-2407-5

[23] Gittins, K., Léna, C.: Upper bounds for Courant-sharp Neumann and Robin eigenvalues. Bull. Soc. Math. France **148**(1), 99–132 (2020). 10.24033/bsmf.2800

[24] Gruber, P.M.: Convex and discrete geometry. Grundlehren der mathematischen Wissenschaften [Fundamental Principles of Mathematical Sciences], vol. 336. Springer, Berlin (2007)

[25] Henrot, A.: Extremum Problems for Eigenvalues of Elliptic Operators. Frontiers in Mathematics. Birkhäuser Verlag, Basel (2006)

[26] Ivriĭ, V.Ja.: The second term of the spectral asymptotics for a Laplace-Beltrami operator on manifolds with boundary. Funktsional. Anal. i Prilozhen. **14**(2), 25–34 (1980)

[27] Kovařík, Hynek: On the lowest eigenvalue of Laplace operators with mixed boundary conditions. J. Geom. Anal. **24**(3), 1509–1525 (2014). 10.1007/s12220-012-9383-4

[28] Kritikos, N.: Über konvexe Flächen und einschließende Kugeln. Math. Ann. **96**(1), 583–586 (1927). 10.1007/BF01209189

[29] Larson, S.: Asymptotic shape optimization for Riesz means of the Dirichlet Laplacian over convex domains. J. Spectr. Theory **9**(3), 857–895 (2019). 10.4171/JST/265

[30] Levitin, M., Mangoubi, D., Polterovich, I.: Topics in spectral geometry, preliminary version dated May 29 (2023). https://michaellevitin.net/Book/

[31] Lotoreichik, V., Rohleder, J.: Eigenvalue inequalities for the Laplacian with mixed boundary conditions. J. Differ. Equ. **263**(1), 491–508 (2017). 10.1016/j.jde.2017.02.043

[32] Li, P., Yau, S.T.: On the Schrödinger equation and the eigenvalue problem. Commun. Math. Phys. **88**(3), 309–318 (1983)

[33] Marshall, N.F.: Stretching convex domains to capture many lattice points. Int. Math. Res. Not. **10**, 2918–2951 (2020). 10.1093/imrn/rny102

[34] Matheron, G.: La formule de Steiner pour les érosions. J. Appl. Probab. **15**(1), 126–135 (1978). 10.2307/3213242

[35] McShane, E.J.: Extension of range of functions. Bull. Am. Math. Soc. **40**(12), 837–842 (1934). 10.1090/S0002-9904-1934-05978-0

[36] Maggi, F., Ponsiglione, M., Pratelli, A.: Quantitative stability in the isodiametric inequality via the isoperimetric inequality. Trans. Am. Math. Soc. **366**(3), 1141–1160 (2014). 10.1090/S0002-9947-2013-06126-0

[37] Netrusov, Y., Safarov, Y.: Weyl asymptotic formula for the Laplacian on domains with rough boundaries. Commun. Math. Phys. **253**(2), 481–509 (2005). 10.1007/s00220-004-1158-8

[38] Payne, L.E., Weinberger, H.F.: An optimal Poincaré inequality for convex domains. Arch. Ration. Mech. Anal. **5**(286–292), 1960 (1960). 10.1007/BF00252910

[39] Ross, M.: The Lipschitz continuity of Neumann eigenvalues on convex domains. Hokkaido Math. J. **33**(2), 369–381 (2004). 10.14492/hokmj/1285766171

